# The C4EB study—Transvamix (10% THC / 5% CBD) to treat chronic pain in epidermolysis bullosa: A protocol for an explorative randomized, placebo controlled, and double blind intervention crossover study

**DOI:** 10.1371/journal.pone.0277512

**Published:** 2022-12-12

**Authors:** Nicholas H. B. Schräder, José C. Duipmans, Remco J. Renken, Peter Sörös, Karin M. Vermeulen, Maria C. Bolling, André P. Wolff

**Affiliations:** 1 Department of Dermatology, University Medical Centre Groningen, University of Groningen, Groningen, The Netherlands; 2 Cognitive Neuroscience Center, Department of Biomedical Sciences of Cells and Systems, University Medical Center Groningen, Groningen, The Netherlands; 3 Department of Neurology, School of Medicine and Health Sciences, Carl von Ossietzky Universität Oldenburg, Oldenburg, Germany; 4 Research Center Neurosensory Science, Carl von Ossietzky University of Oldenburg, Oldenburg, Germany; 5 Department of Epidemiology, University Medical Centre Groningen, University of Groningen, Groningen, The Netherlands; 6 Anaesthesiology Pain Centre, University Medical Centre Groningen, University of Groningen, Groningen, The Netherlands; University of Texas Medical Branch at Galveston, UNITED STATES

## Abstract

Patients with the genetic blistering skin condition epidermolysis bullosa (EB) report severe pain as a consequence of skin and mucous membrane lesions including blisters, wounds, and scars. Adequate symptom alleviation is not often achieved using conventional pharmacologic interventions. Finding novel approaches to pain care in EB is imperative to improve the quality of life of patients living with EB. There are several anecdotal reports on the use of cannabinoid-based medicines (CBMs) by EB patients to reduce the burden of symptoms. However, controlled clinical investigations assessing these reported effects are lacking. As the pain quality “unpleasantness” delineates EB pain, we hypothesize the modulation of affective pain processing in the brain by way of intervention with CBMs comprising the cannabinoids Δ-9-tetrahydrocannabinol and cannabidiol—objectified by functional magnetic resonance imaging (fMRI). The C4EB study is an investigator-initiated, single-centre, randomized, double-blind, placebo-controlled and crossover trial. Adult patients with the diagnosis epidermolysis bullosa, reporting chronic pain will be eligible to participate. Following baseline measurements, participants will be randomized to receive the sublingually administered interventions placebo and Transvamix^®^ in forward or reversed orders, each for two weeks and separated by a washout. The primary outcome is the difference in numeric rating scale pain scores between grouped interventions, using affective descriptors within the Short-form McGill Pain Questionnaire-2. Secondary outcomes include pain self-efficacy, concomitant analgesic medication-use and adverse events. Additionally, fMRI will be employed to assess brain connectivity related to neuroanatomic pain circuits at baseline, placebo and Transvamix^®^ interventions. The study was approved by the ethical committee at the University Medical Center of Groningen in the Netherlands. Results will be submitted for publication in a peer-reviewed journal.

**Trial registration number**: Netherlands Trial Register: NL9347 (Acronym: C4EB).

## Introduction

Patients with the genetic blistering skin condition, epidermolysis bullosa (EB), report a significant impact on quality of life compared to other skin conditions [1]. EB is considered a rare disease with an incidence and point-prevalence in the Netherlands of 41.3 per million live births and 22.4 per million population, respectively [2]. Amongst adults with EB, pain is the most debilitating symptom initially caused by blistering, chronic wounds, skin contractures and neuropathies [3]. Persistent pain in EB becomes a complex multidimensional experience due to psychosocial, and emotional components [3–6]. Due to this complex nature of pain in EB, adequate analgesia is difficult to achieve, leading to chronic refractory pain. In a recent cross-sectional study on EB in the Netherlands, patients with all EB-types rated “unpleasant” pain quality as the highest score of 20 qualities of pain [7]. This is interesting as research has shown that pain affect, located in the limbic system, which can be reported as the pain quality “unpleasantness”, is functionally distinct from sensory discriminative pain processing pathways [8]. One method to assess pain affect is by using functional magnetic resonance imaging (fMRI) techniques to measure brain connectivity in known and suspected neuroanatomical areas processing pain affect [8–10]. Research groups applying this technique have identified the amygdala and anterior cingulate cortex (ACC) as central brain areas with altered connectivity during chronic pain. Furthermore, independent of the aetiology of chronic pain, all chronic pain states (measured by fMRI) show similarities in altered brain connectivity, sometimes referred to as neuronal reorganization, which can be subsequently reversed with effective treatments [9].

Endogenously produced cannabinoids (endocannabinoids) play a key role in the modulation of pain signals by binding endocannabinoid receptors 1 and 2 (CB1/2) [11, 12]. CB1/2 are ubiquitous in the human body, however CB1 is most prevalently found in the central nervous system with the exception of lower brain-stem regions for cardiovascular and respiratory functions—explaining the unattainable lethal doses and complete absence of mortality from exogenous plant-derived cannabinoid (phytocannabinoid) administration [13–15]. As CB1/2 are densely populated in the frontal-limbic areas of the brain, their role in modulating pain affect has been proposed [16]. Like these endocannabinoids, exogenous cannabinoids, namely delta-9-tetrahydrocannabinol (THC) and cannabidiol (CBD), also target these receptors, modulating neurotransmitter release in the CNS. Specifically, the amygdala and ACC are shown to have altered functional connectivity to brain cortices through CB1/2 agonism [16]. This change in connectivity significantly correlates with a decrease in pain, and more specifically, a decrease in the pain quality “unpleasantness” from noxious stimuli after systemic administration of phytocannabinoids [16–18]. In recent years, clinical studies on various painful diseases have shown the superiority of combining multiple cannabinoids compared to single-cannabinoid extracts, with specific regard to the combination of THC and CBD [19–21]. This is likely attributed to an improved tolerance of THC-based interventions, by way of paradoxical CBD CB1/2 receptor antagonism [20, 21].

It is important to note that the use of cannabinoid-based medicines (CBM) for pain, often as self-medication, have preceded clinical studies and sufficient clinical evidence. Given the limited effectiveness of conventional treatments in EB, it is well-known that these patients have used CBMs for symptom alleviation and have subsequently reported promising anecdotes. A recent study collected data on 71 EB patients around the world using CBMs. Although the cohort mainly comprised patients with a more severe EB, all EB-types described symptomatic improvements. A considerable obstacle to interpreting these experiences is the significant variability in administration forms, cannabinoid compositions, and doses used by patients. Additionally, these products are often not regulated as pharmaceutical products and therefore lack pharmaceutical-grade standardisation and quality control. However, seventeen of the participants administered CBMs sublingually and retrospectively reported mean NRS (0–10) pain reductions of 3.58 (±1.8) after administering CBMs [22].

In the Netherlands, CBMs are produced and compounded by companies overseen by the Dutch government and have been available on prescription since 2003. From 2015 CBM oils comprising THC and CBD in varying ratios have been empirically prescribed to several Dutch patients with EB [23]. Good quality evidence on CBM effectiveness in EB, and mechanisms of action, remain elusive. Nevertheless, clinical studies on heterogenous chronic pain conditions show moderate evidence for the efficacy of CBMs (comprising THC and CBD) versus placebo [24, 25].

To date, it has also been shown that neither the dose administered nor detected plasma levels of cannabinoids are good predictors of pain alleviation when treated with CBMs. The high inter-individual variability (CV%) is likely due to patient variations in BMI, enterohepatic circulation, degradation in the gut, previous cannabinoid exposure, the endogenous cannabinoid system (endocannabinoid system) compositions and differences in administration technique [26, 27].

Therefore, the current best-practice is for patients administering CBMs to undergo dose-escalation titration following a premeditated dosage build-up scheme (e.g., administration 4x daily). In order to achieve an effective dose, patients continue titration until side-effects are reached, then reduce their dose by one step in the titration scheme, and subsequently maintain the achieved dose [28]. Methods for dose-escalation vary significantly between patients in clinical practice, which is why a fixed titration scheme should be employed for research studies. This feasibly standardizes the dose-escalation for all participants in clinical studies, individualizing the dose-required but standardizing the method to achieve this. Controlled studies in rare diseases, like EB, encounter several hurdles including small population sizes, high participation burden and ethical dilemmas related to placebo administration. To address these, the proposed study allows for participation with unchanged medication regimens already in place and employs a crossover methodology where participants will receive both placebo and the chemically active intervention for a short period of time.

Taken together, the unmet treatment needs for pain in EB and moderate quality evidence for the use of CBMs to treat pain and modulate pain affect, open grounds for further clinical investigation. To substantiate this research avenue, CBM interventions in EB, the proposed explorative study will gain insight into effects of Transvamix^®^, a sublingually administered and standardized CBM, on pain, brain connectivity, doses achieved through titration and adverse events. New fMRI techniques to assess brain connectivity enhance the assessment of both baseline connectivity characteristics in EB patients with pain, as well as connectivity changes in areas processing pain affect. In the clinical research setting it is therefore imperative to standardize dose-escalation schemes, administer standardized pharmaceutical grade CBMs, apply validated patient reported outcomes, and employ sufficient sample sizes and rigorous statistical analyses. We hypothesize a novel avenue of pain intervention for EB by modulating pain affect–objectified by fMRI.

## Methods and analysis

### Design and setting

The study design is an explorative, randomized placebo-controlled and double-blind intervention crossover study: Transvamix^®^ (100mg/mL THC / 50mg/mL CBD) to treat chronic pain in Epidermolysis Bullosa. This is an explorative study because cannabinoid-based medicines (CBMs), Transvamix-like drugs, have not yet been studied by way of controlled trials in the EB patient population. The addition of placebo mitigates confounding participation bias. The crossover design reduces the number of participants required, an important factor when studying rare diseases, and supported by randomization and blinding, enables rigorous statistical analyses. In addition to this, effects of group outcomes on a group level can be analysed. Participant inclusion will commence in Q4 of 2022 for a period of 6 months. Study completion is expected in Q4 of 2023. After completion, analysis will be performed whereby results publication is expected in Q4 of 2023.

### Inclusion, exclusion and randomization

Participants in this study are adult patients with a confirmed diagnosis (genetic testing, immunofluorescent diagnostics, or electron microscopy) of inherited epidermolysis bullosa (EB). All patients with EB in the Netherlands are registered at the Dutch Expertise Centre for Blistering Diseases, Department of Dermatology, University Medical Centre Groningen. To participate, patients must be currently living in the Netherlands and are at least 16 years of age. Participation is not limited to one sex, nor to any ethnic group. Written informed consent will be obtained and stored in a locked cabinet at the research centre.

Eligible participants, who have consented to participation, will undergo inclusion screening at the research centre. Subjects must meet all the following criteria (Table 1). The Trial Master File will be maintained by Felix Farma B.V., who will also manage blinding of participant data, as well as randomization and allocation. An a priori randomization code generator will be completed before the start of inclusion, for the total expected number of participants. Participants will be allocated to groups receiving intervention in the orders T-P or P-T (T = Transvamix; P = Placebo). Confirmation of participant inclusion will be recorded in the eCRF. Either Transvamix^®^ or placebo will be delivered by courier to participants 3 days before the allocated commencement of treatment administration. Instructions for administration are provided both verbally and in writing to participants.

**Table 1:** Inclusion and exclusion criteria.

**Inclusion criteria**
1. Clinical diagnosis, supplemented by genetic analysis, immunofluorescent diagnostics, or electron microscopy of congenital epidermolysis bullosa (EB).
2. At least 16 years of age from the date inclusion.
3. Can read and write in the Dutch language.
4. Mentally competent and legally able to appreciate informed consent.
5. Reporting an average pain mean score ≥4 on NRS (0–10) averaged throughout the previous week at one of the following times of day: morning, afternoon, or evening.
**Exclusion criteria**
1. Patients enrolled in other clinical trials that do not allow for a deviation in treatment.
2. Have experienced myocardial infarction or clinically significant cardiac dysfunction within the last 12 months or have had a cardiac disorder that, in the opinion of the investigator would have put the participant at risk of a clinically significant arrhythmia or myocardial infarction.
3. Patients with known psychotic disorder (including the use of antipsychotic medications), or a history of suicidal ideation.
4. Female patients of child-bearing potential and male participants whose partner was of child-bearing potential, unless willing to ensure that they or their partner used effective contraception.
5. Patients who have had significantly impaired renal or hepatic function in the last 12 months.
6. The patient is currently using or has used cannabis or cannabinoid-based medications within 30 days of study entry and was unwilling to abstain for the duration of the study.
7. Patients unwilling or unable to refrain from driving road vehicles and/or using potentially dangerous machinery where sufficient concentration is necessary, during the study period.
8. Patients unable to stay within the Netherlands for the duration of the study period.
9. History of addiction and/or hospital admission due to addiction to recreational or pharmaceutical drugs.
10. Patients with contraindications for MRI determined using the MRI safety form pertaining to for example, surgical, cosmetic, or accident-related metal objects in/on the body; claustrophobia.

### Intervention: Transvamix^®^ and placebo

The investigational medicinal product (IMP) is Transvamix^®^, a cannabinoid-based medicine (CBM) comprising 100mg/mL THC (*delta-9-tetrahydrocannabinol*) and 50mg/mL CBD (*cannabidiol*) (*Transvamix-oil THC 10% / CBD 5%*, *10ml*). Transvamix^®^ is prepared by Felix Farma B.V. using pharmaceutical-grade cannabis produced by Bedrocan B.V., and isolated using an ethanol-based extraction process. The product, Transvamix^®^, has a standardized cannabinoid composition within regulatory margins. The placebo formulation is comparable in texture, colour, and taste. Identical packaging and labelling will be used. Both Transvamix^®^ and placebo will be administered using a 1 ml dosing syringe.

**Fig 1:**
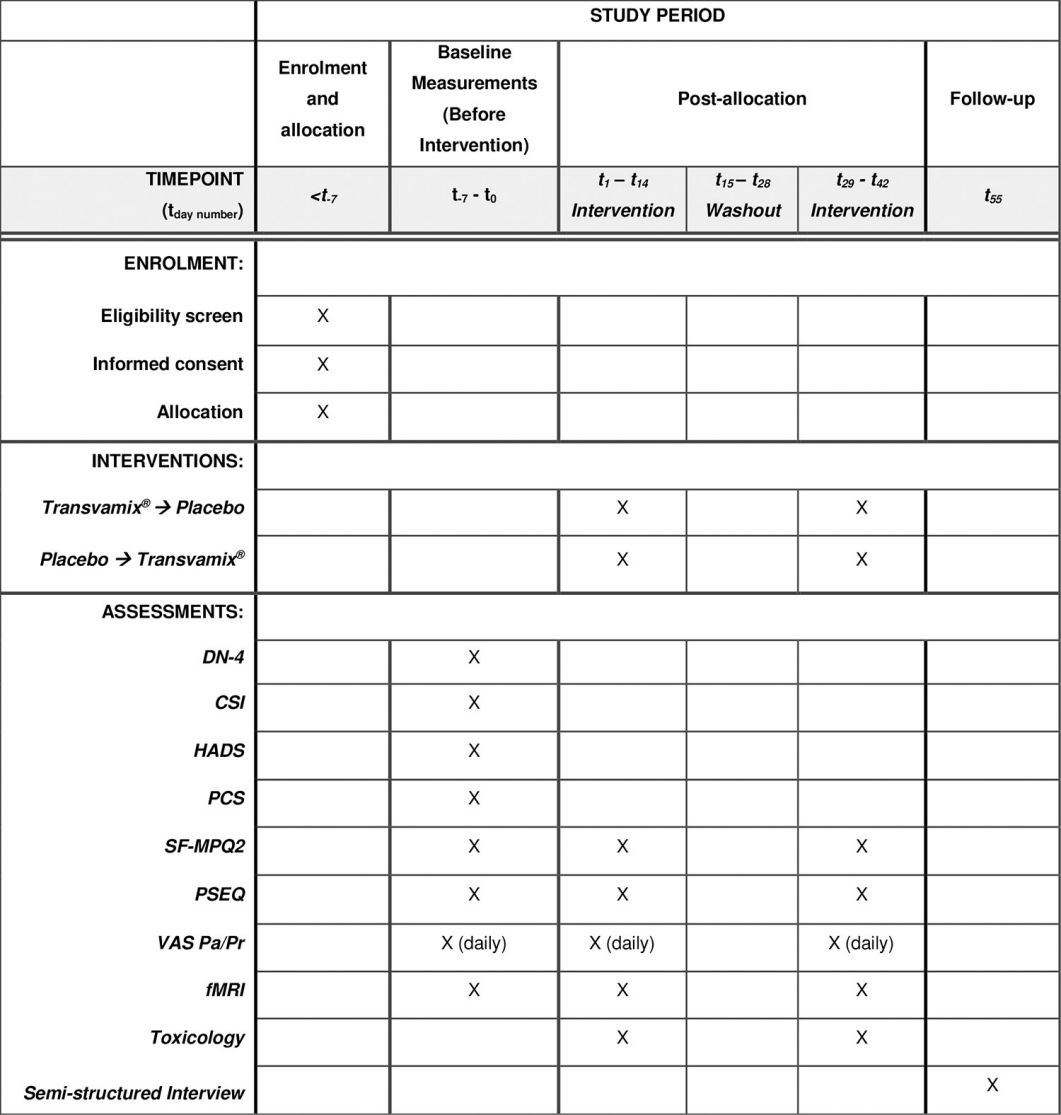
Schedule of enrolment, interventions, and assessments (SPIRIT figure). Participation duration: 64 days, or 9 weeks and 1 day. Duration of participant exposure to interventions: 14 days (placebo), 14 days (Transvamix^*®*^). DN-4: douleur neuropathique 4, CSI: central sensitization inventory, PCS: pain catastrophizing scale, SF-MPQ2: short form McGill pain questionnaire 2, PSEQ: pain self-efficacy questionnaire, VAS Pa/Pr: visual analogue scale pain/pruritus, fMRI: functional magnetic resonance imaging.

Participants will receive both the IMP (Transvamix^®^) and placebo in forward (T→P) or reverse (P→T) orders, depending on treatment arm allocation after randomization (Fig 1). The study drugs (Transvamix^®^ and placebo) will be provided by Felix Farma B.V. A washout of two weeks between both interventions, based on cannabinoid metabolite half-lives, will mitigate carry-over effects.

All participants will follow the predetermined titration scheme (Table 2). The titration will be stopped at the onset of side-effects (as reported by the participant), and the dose administered reduced by one day in the titration scheme. This will be called the sub-side effect threshold maintenance dose.

**Table 2:** Titration scheme employed by study participants.

Transvamix^*®*^ (100mg/mL THC and 50mg/mL CBD) and Placebo					Total (ml)	Total THC (mg)	Total CBD (mg)
Day	06:00–09:00	11:00–14:00	16:00–19:00	21:00–00:00			
1	-	-	-	0,05 ml	0,05	5	2,5
2	-	-	-	0,05 ml	0,05	5	2,5
3	0,05 ml	-	-	0,05 ml	0,1	10	5
4	0,05 ml	-	0,05 ml	0,05 ml	0,15	15	7,5
5	0,05 ml	0,05 ml	0,05 ml	0,05 ml	0,2	20	10
6	0,05 ml	0,05 ml	0,05 ml	0,1 ml	0,25	25	12,5
7	0,1 ml	0,05 ml	0,05 ml	0,1 ml	0,3	30	15
8	0,1 ml	0,05 ml	0,1 ml	0,1 ml	0,35	35	17,5
9	0,1 ml	0,1 ml	0,1 ml	0,1 ml	0,4	40	20
10	0,1 ml	0,1 ml	0,1 ml	0,15 ml	0,45	45	22,5
11	0,15 ml	0,1 ml	0,1 ml	0,15 ml	0,5	50	25
12	0,15 ml	0,1 ml	0,15 ml	0,15 ml	0,55	55	27,5
13	0,15 ml	0,15 ml	0,15 ml	0,2 ml	0,65	65	32,5
14	0,2 ml	0,15 ml	0,2 ml	0,2 ml	0,75	75	37,5

This achieved dose will be maintained for the remaining intervention period until day 14 is completed for each intervention (Transvamix^®^ and placebo). Both placebo and IMP (Transvamix^®^) will undergo the same titration scheme. The maximum dose is 0.75ml/day Transvamix^®^ or placebo (divided between 4 separate administrations). See Table 2 for the titration period.

### Drug safety

Transvamix^®^ is produced by the Transvaal pharmacy as a compounded medication and prescribed to patients with refractory symptoms, of which pain is the most prevalent indication. CBM-oil prescriptions have been filled out over 100,000 times to patients in the Netherlands between 2015 and 2020 [29]. Since their appearance on the market 52 adverse events related to sublingually administered CBMs (of which 1 was serious: hypoglycaemia) have been reported to the centre for pharmacovigilance (LAREB) [30].

### Safety monitoring

Although risk for serious adverse events is estimated to be low, a data safety monitoring board (DSMB) will be compiled, consisting of four independent members with expertise in: clinical pharmacology, longitudinal research statistics, EB clinical care, and pain/cannabinoid research. The DSMB will maintain an overview of (serious) adverse events and offer independent advice for premature completion or termination of the study.

### Care as usual

Participants will continue treatments, including analgesic regimens, according to their current prescriptions and EB breakthrough pain guidelines [3]–which include instructions for breakthrough symptoms. The IMP is not intended for breakthrough symptoms as participants will adhere to a strict dosing regimen. During the baseline visit participants will report all currently used systemic treatments. Participants will report changes in dosing or additional administration to these medications (for example, breakthrough medication) in the participant’s logbook.

### Follow-up and data collection

Study data will be collected and managed using REDCap electronic data capture tools hosted at the research centre [31, 32]. REDCap (Research Electronic Data Capture) is a secure, web-based software platform designed to support data capture for research studies. Participants will receive URLs to access the surveys for online completion from a smartphone, tablet, or computer.

Subjects can leave the study at any time for any reason if they wish to do so without any consequences. The investigator can decide to withdraw a subject from the study for urgent medical reasons or non-compliance.

### Primary outcome measures

The primary outcome is the change score for numeric rating scale (NRS) values using the affective descriptors from the short-form McGill Pain Questionnaire 2 (SF-MPQ2). This will be measured as the grouped difference in mean NRS scores pre- (baseline) and post- intervention (Transvamix^®^ vs. placebo).

### Secondary outcome measures

The secondary outcomes will include data derived from other patient reported outcome measurements (PROM). These outcomes will be measured as grouped differences, change scores, in mean pre- (baseline) and post-intervention (Transvamix^®^ vs. placebo) scores. The instruments used are the SF-MPQ2 and Pain Self Efficacy Questionnaire (PSEQ) comprising NRS values. Additionally, change scores for global pain and pruritus will be measured using visual analogue scales (VAS 0–100).

Functional MRI data pertaining to brain connectivity will be analysed between placebo and Transvamix^®^ groups. The frequency and participant reported burden (NRS 0–10, where 0 is “no burden whatsoever”, and 10 is the “highest burden possible”) of side-effects will be reported and compared between Transvamix^®^ and placebo.

Lastly, analgesic medication changes, of currently used medications reported at baseline, will be reported by participants at the end of each intervention. Reductions in- or increased use will be quantified and compared between intervention groups.

### MRI data acquisition

Whole-brain fMRI blood oxygen-level dependent data will be acquired on a 3T Siemens Magnetom Prisma with a 64-channel head coil at the Cognitive Neuroscience Centre in Groningen (https://bscs.umcg.nl/nl/faciliteiten/cognitive-neuroscience-center/), using a multi-echo echo-planar T2*-weighted imaging sequence with 550 volumes (voxel size of 2.5 × 2.5 × 2.5 mm^3^ [33]. A high-resolution T1-weighted anatomical image will also be obtained (voxel size: 1×1×1 mm^3^). An experienced MRI technologist will examine all structural images to ensure the absence of structural abnormalities in the brain. In case of an abnormality, the participant will be referred to their general practitioner for further examination or referral.

### Sample size

We performed a sample size calculation based on data (n = 28) from a recent study in Dutch patients with EB who had pain scores >3. In 28 patients, mean NRS pain scores were 5.76 ± 1.45 [34].

- To establish a significant reduction in pain scores of 30%, with a power of 80% and a 0.05, the minimum sample size is 9 (or 5 participants in each study arm)
- To establish a significant reduction in pain scores of 50%, the minimum sample size is 5 (or 3 participants in each study arm).

Owing to the rarity of EB and the strict inclusion criteria for age and chronic pain, the estimated feasible participant recruitment number is 16. For this study we aim to include 16 participants (8 in each study arm).

### Data handling

All data will be recorded in the eCRF REDCap, completed by either the researcher or participant, when prompted. Data will be handled confidentially and accordingly to the European guidelines for privacy protection (AVG). Research participants will be pseudonymized, personally traceable data removed, and will be identified using a participation number. Data will be stored for a minimum of 15 years after study closure.

### Data analysis

Primary and secondary outcomes will be presented using descriptive statistics (means and standard deviations, or median and interquartile ranges), and subdivided into three groups (baseline, placebo and Transvamix^®^). The change scores for baseline to placebo and Transvamix^®^ will be computed and incorporated for statistical testing. Visual inspection of data and a Shapiro Wilk test will be used to check for normality. Next either t-testing (paired and independent samples t-testing) or non-parametric tests (including Wilcoxon signed rank test) will be used depending on the distribution observed. The significance level will be set to p<0.05. In addition, mixed effects models will be applied, allowing for analysis of longitudinal data, missing data, and possible period effects.

The resting-state multi-echo fMRI dataset will be preprocessed using the ME-ICA pipeline (https://me-ica.readthedocs.io/en/latest/) [33, 35]. ME-ICA uses multi-echo independent component analysis to perform motion correction and slice-time correction of the original dataset. The resulting preprocessed datasets will then be analyzed using the Matlab-based CONN toolbox (https://web.conn-toolbox.org; RRID:SCR_009550) [36]. To study the effects of Transvamix^®^ we will primarily perform a seed-based correlation analysis, to determine the functional connectivity between the seed regions, the anterior cingulate cortex (ACC) and amygdala, and other areas in the brain. Changes in brain connectivity (baseline vs. Transvamix^®^ vs. placebo) will be explored in individual participants and on the group level. The neural effects of the intervention will be correlated against primary and secondary outcome measures.

### Ethical compliance

The methods employed in this trial were judged and approved by the Central Committee on Research Involving Human Subjects (*Centrale Commissie Mensgebonden Onderzoek*, CCMO, the National Medical Ethical Committee in the Netherlands). The EudraCT number is 2021-000103-20. The trial was registered prospectively in the Netherlands Trial Register (NL9347). Changes to the study protocol will be submitted to the CCMO in amendments for approval.

### Public involvement and funding

This is a study supported by the patient organization Dystrophic Epidermolysis Research Association United Kingdom (DEBRA-UK).

## Conclusion

In summary this explorative study will assess the use of pharmaceutical grade CBMs comprising THC and CBD in patients with EB suffering from chronic pain. The controlled cross over design of the study minimizes the burden of participation and maximizes the methodological reproducibility by employing standardized interventions and objective research outcomes. Both the chosen methodology and eventual results highlight a novel research avenue and will cornerstone future clinical research in EB and CBMs.

## Supporting information

S1 Checklist SPIRIT 2013 checklist: Recommended items to address in a clinical trial protocol and related documents*. (DOC)

S1 Protocol (PDF)
