# Supplementary material for: The C4EB study—Transvamix (10% THC / 5% CBD) to treat chronic pain in epidermolysis bullosa: A protocol for an explorative randomized, placebo controlled, and double blind intervention crossover study
Source: PLoS One. 2022 Dec 12;17(12):e0277512. doi: 10.1371/journal.pone.0277512 (PMC9744305; doi:10.1371/journal.pone.0277512)
Supplement: S1 Protocol — (PDF) [file pone.0277512.s002.pdf]

RESEARCH PROTOCOL

Transvamix (10% THC / 5% CBD) to treat chronic pain in Epidermolysis Bullosa: An explorative randomized, placebo-controlled and double-blind intervention crossover study

The C4EB study

Protocol Title: Transvamix (10% THC / 5% CBD) to treat chronic pain in Epidermolysis Bullosa: An explorative randomized, placebo-controlled and double-blind intervention crossover study

**Protocol ID**	**202000638**<br>**NL Trial Register: NL9347**
**Short title**	**C4EB**
**EudraCT number**	*2021-000103-20*
**Version**	Version 2.0
**Date**	**21-01-2020**
**Principal investigator(s) (in Dutch: hoofdonderzoeker/ uitvoerder)**	Prof. André P Wolff (a.p.wolff@umcg.nl)<br>Dr. Marieke C Bolling (m.c.bolling@umcg.nl)
**Sponsor (in Dutch: verrichter/opdrachtgever)**	University Medical Center Groningen Hanzeplein 1<br>P.O. Box 30.001<br>9700 RB Groningen<br>The Netherlands
**Subsidising party**	DEBRA-UK
**Independent expert (s)**	Drs. Bouwe Molenbuur – Anesthesioloog / Multidisciplinaire team voor epidermolysis bullosa. (b.molenbuur@umcg.nl)
**Pharmacy**<br><br><br>**IMP producer**	**Transvaal Apotheek**<br>Kempstraat 113 2572 GC Den Haag Tel. +3170-3469314<br><br>**Felix Farma**<br>Kempstraat 113 2572 GC Den Haag<br>info@felixfarma.nl

PROTOCOL SIGNATURE SHEET

Name	Signature	Date
**Sponsor or legal representative:**<br>***Prof. dr. André P Wolff***<br>***head of department, anesthesiology pain centre, UMC Groningen.***		
**Principal Investigator:**<br>***Prof. dr. André P Wolff***		

C4EB study v2 NL76471.042.21 METc: 202000638

Protocol Title: Transvamix (10% THC / 5% CBD) to treat chronic pain in Epidermolysis Bullosa: An explorative randomized, placebo-controlled and double-blind intervention crossover study

**Protocol ID**	**202000638**<br>**NL Trial Register: NL9347**
**Short title**	**C4EB**
**EudraCT number**	*2021-000103-20*
**Version**	Version 2~~1~~.0
**Date**	**21-01-2020**
**Principal investigator(s) (in Dutch: hoofdonderzoeker/ uitvoerder)**	Prof. André P Wolff (a.p.wolff@umcg.nl)<br>Dr. Marieke C Bolling (m.c.bolling@umcg.nl)
**Sponsor (in Dutch: verrichter/opdrachtgever)**	University Medical Center Groningen Hanzeplein 1<br>P.O. Box 30.001<br>9700 RB Groningen<br>The Netherlands
**Subsidising party**	DEBRA-UK
**Independent expert (s)**	Drs. Bouwe Molenbuur – Anesthesioloog / Multidisciplinaire team voor epidermolysis bullosa. (b.molenbuur@umcg.nl)
**Pharmacy**	**Transvaal Apotheek**<br>Kempstraat 113 2572 GC Den Haag Tel. +3170-3469314
**IMP producer**	**Felix Farma**<br>Kempstraat 113 2572 GC Den Haag<br>info@felixfarma.nl

C4EB study v2 NL76471.042.21 METc: 202000638

PROTOCOL SIGNATURE SHEET

Name	Signature	Date
**Sponsor or legal representative:**<br>*Prof. dr. André P Wolff*<br>*head of department, anesthesiology pain centre, UMC Groningen.*		7-4-'21
**Principal Investigator:**<br>*Prof. dr. André P Wolff*		7-4-'21

1. INTRODUCTION AND RATIONALE **11**

1.1 General Rationale 11

1.2 Transvamix-oil (cannabinoid-based medicine oil) 11

1.3 Transvamix & Pain in EB 12

1.4 Concluding Remarks 14

2. OBJECTIVES **15**

2.1 Hypothesis: 15

2.2 Primary Objective 15

2.3 Secondary Objectives 15

3. STUDY DESIGN **16**

4. STUDY POPULATION **18**

4.1 Population (base) 18

4.2 Inclusion criteria 18

4.3 Exclusion criteria 18

4.4 Sample size calculation 19

5. TREATMENT OF SUBJECTS **20**

5.1 Investigational product/treatment 20

5.2 Escape medication 20

6. INVESTIGATIONAL PRODUCT **21**

6.1 Name and description of investigational product(s) 21

6.2 Summary of findings from non-clinical studies 21

6.3 Summary of findings from clinical studies 21

6.4 Summary of known and potential risks and benefits 21

6.5 Description and justification of route of administration and dosage 21

6.6 Dosages, dosage modifications and method of administration 22

6.7 Preparation and labelling of Investigational Medicinal Product 23

6.8 Drug accountability 24

7. NON-INVESTIGATIONAL PRODUCT **25**

8. METHODS **26**

8.1 Study parameters/endpoints 26

8.2 Randomisation, blinding and treatment allocation 27

8.3 Study procedures 27

8.4 Withdrawal of individual subjects 29

8.5 Replacement of individual subjects after withdrawal 30

8.6 Follow-up of subjects withdrawn from treatment 30

8.7 Premature termination of the study 30

9. SAFETY REPORTING 31

9.1 Temporary halt for reasons of subject safety 31

9.2 AEs, SAEs and SUSARs 31

9.3 Annual safety report 33

9.4 Follow-up of adverse events 33

9.5 Data Safety Monitoring Board (DSMB) / Safety Committee 33

10. STATISTICAL ANALYSIS 34

10.1 Primary study parameter(s) 34

10.2 Secondary study parameter(s) 34

10.3 Other study parameters 35

10.4 Interim analysis (if applicable) 35

11. ETHICAL CONSIDERATIONS 36

11.1 Regulation statement 36

11.2 Recruitment and consent 36

11.3 Objection by minors or incapacitated subjects (if applicable) 36

11.4 Benefits and risks assessment, group relatedness 36

11.5 Compensation for injury 37

11.6 Incentives 37

ADMINISTRATIVE ASPECTS, MONITORING AND PUBLICATION 38

12. Handling and storage of data and documents 38

12.1 Monitoring and Quality Assurance 38

12.2 Amendments 38

12.3 Annual progress report 39

12.4 Temporary halt and (prematurely) end of study report 39

12.5 Public disclosure and publication policy 39

13. STRUCTURED RISK ANALYSIS 40

13.1 Potential issues of concern 40

13.2 Synthesis 43

14. REFERENCES 44

LIST OF ABBREVIATIONS AND RELEVANT DEFINITIONS

**ACC**	Anterior Cingulate Cortex
**AE**	Adverse Event
**AR**	Adverse Reaction
**BOLD**	Blood Oxygen Level Dependent Imaging
**CA**	Competent Authority
**CB1/2**	Cannabinoid-binding receptors 1 and 2
**CBD**	Cannabidiol (cannabinoid)
**CCMO**	Central Committee on Research Involving Human Subjects; in Dutch: Centrale Commissie Mensgebonden Onderzoek
**CBM**	Cannabinoid-based Medicine
**CV**	Curriculum Vitae
**CSI**	Central sensitization index
**DN-4**	Douleur Neuropathique 4
**DSMB**	Data Safety Monitoring Board
**EB**	Epidermolysis Bullosa
**EU**	European Union
**EudraCT**	European drug regulatory affairs Clinical Trials
**fMRI** (BOLD)	Functional Magnetic Resonance Imaging (Blood Oxygen Level Dependent)
**GCP**	Good Clinical Practice
**GDPR**	General Data Protection Regulation; in Dutch: Algemene Verordening Gegevensbescherming (AVG)
**HADS**	Hospital anxiety and depression survey
**IB**	Investigator's Brochure
**IC**	Informed Consent
**IMP**	Investigational Medicinal Product
**IMPD**	Investigational Medicinal Product Dossier
**METC**	Medical research ethics committee (MREC); in Dutch: medisch-ethische toetsingscommissie (METC)
**SF-MPQ2**	Short-form McGill Pain Questionnaire 2
**PCS**	Pain catastrophizing scale
**PSEQ**	Pain self efficacy questionnaire
**(S)AE**	(Serious) Adverse Event
**SPC**	Summary of Product Characteristics; in Dutch: officiële productinformatie IB1-tekst
**Sponsor**	The sponsor is the party that commissions the organisation or performance of the research, for example a pharmaceutical

	company, academic hospital, scientific organisation or investigator. A party that provides funding for a study but does not commission it is not regarded as the sponsor, but referred to as a subsidising party.
**SUSAR**	Suspected Unexpected Serious Adverse Reaction
**THC**	Delta-9-Tetrahydrocannabinol (cannabinoid)
**UAVG**	Dutch Act on Implementation of the General Data Protection Regulation; in Dutch: Uitvoeringswet AVG
**WMO**	Medical Research Involving Human Subjects Act; in Dutch: Wet Medisch-wetenschappelijk Onderzoek met Mensen

SUMMARY

Rationale: There is an unmet need for more effective pain alleviation in EB patients. EB patient anecdotes on the use of cannabinoid-based medicines (CBMs) are in line with current science aimed at assessing the effectiveness of CBMs for chronic pain conditions. Until now evidence on the effectiveness of CBMs is moderate and is inconclusive. As the pain quality item “unpleasantness” delineates EB pain, we hypothesize the modulation of affective pain processing in the brain by way of intervention with Transvamix (a CBM comprising THC and CBD) - objectified by functional magnetic resonance imaging (fMRI).

Objective: The primary objective is to determine the effect of Transvamix on the pain quality item “unpleasantness”, relative to placebo. Secondary objectives include the assessment of general pain qualities, pain self-efficacy, general pain and pruritus intensity, functional brain connectivity, adverse events and sub-side effect threshold maintenance dose achieved during titration.

Study design: The study is a two-arm, randomized, double-blind, placebo controlled, crossover intervention study.

Study population: Sixteen mentally competent patients with EB, 16 years or older, registered at the Centre for Blistering Diseases (department of Dermatology, UMCG) with a genetic diagnosis of EB – and an average pain score >3 on a numeric rating scale (NRS 0-10).

Intervention: Transvamix-oil comprising 100mg/mL THC and 50mg/mL CBD (10% THC / 5% CBD), and placebo, to be administered sublingually by a 1mL dosing syringe.

Main study parameters/endpoints: The effect of Transvamix, relative to placebo measured by a reduction in the pain quality items for “unpleasantness” (using the short-form McGill pain questionnaire by 30% and 50%; after 2 weeks of Transvamix and 2 weeks of placebo.

Nature and extent of the burden and risks associated with participation, benefit and group relatedness: Currently epidermolysis bullosa patients are using cannabinoid-based medicines both with and without medical supervision. Participants are 16 years or older and mentally competent. They will receive the study intervention Transvamix-oil, a compounded medication *(magistrale bereiding)* readily available on prescription and is currently used by patients for various medical indications in the Netherlands. Transvamix-oil can produce side effects that can be discomforting, but are mild, well tolerated, and disappear within 4-8 hours after administration upon which the following dose can be reduced to mitigate the recurrence of side-effects. Participants will undergo 3x 30 minute MRI scans. A daily logbook will be completed at home electronically - pertaining to the dose administered, possible side-effects and pain/pruritus scores. All participant reported outcomes are completed electronically at home.

1. INTRODUCTION AND RATIONALE

1.1 General Rationale

Patients with the inheritable blistering disease, epidermolysis bullosa (EB), report a significant impact on quality of life compared to other skin conditions [1]. Amongst adults with EB, pain is the most debilitating symptom caused by blistering, chronic wounds, skin contractures and neuropathy. Since 2015 a cannabinoid-based medicine (CBM) oil comprising *Δ9-tetrahydrocannabinol* (THC) and *cannabidiol* (CBD) has been empirically prescribed to Dutch patients with EB, with a reported alleviation of pain, pruritus and reduction in the use of concomitant medications [2]. A very recent study (publication expected April 2021 - Schräder *et al*) collected data on 71 EB patients around the world using CBMs. Seventeen participants administered CBMs sublingually and reported a mean NRS (0-10) pain reduction of 3.58 (±1.8) prior to and after administering CBMs. To date, no clinical trials have studied the effectiveness of sublingually administered CBMs to treat pain in EB patients [3]. In order to substantiate this research avenue, CBM interventions in EB, the proposed explorative study will gain insight into effects of Transvamix, a pharmaceutical grade CBM, on pain, pruritus, brain connectivity, doses achieved through titration and adverse events.

1.2 Transvamix-oil (cannabinoid-based medicine oil)

Transvamix-oil is produced by the Transvaal pharmacy as a compounded medication (*magistrale bereiding*) and prescribed to patients with refractory symptoms, of which pain is the most prevalent indication. CBM-oil prescriptions have been filled out over 100,000 times to patients in the Netherlands between 2015 and 2020 [4]. Since their appearance on the market 52 adverse events related to sublingually administered CBMs (of which 1 was serious: hypoglycemia) have been reported to the center for pharmacovigilance (LAREB). Activities such as driving vehicles or using dangerous apparatus can be impaired in the acute phase after administering CBMs containing both THC and CBD, such as through delayed reaction times. A recent study highlighted that after a single administration of 13.75mg THC and CBD, which was vaporized and inhaled, the resulting impairments did not exceed 240 minutes.[5] Additionally, a placebo-controlled crossover study using an oromucosal THC:CBD spray in varying compositions, observed the largest decrease in subjective wakefulness at 3 hours post administration.[6]

In general, short-term use of Transvamix-like drugs increases the risk for non-serious adverse events 1.86 times, of which dizziness is the most frequently reported [7]. The side-effect profile however is described as acceptable for clinical practice. Detectable plasma

THC levels after oromucosal absorption are reached at 30-45 minutes and maximum concentration at 2-3 hours [8]. Increasing dose tolerance to therapeutic effects has not been observed in Transvamix-like drugs [9].

1.3 Transvamix & Pain in EB

The use of CBMs as treatments for pain have preceded scientific studies (such as phase I and II studies) whereby great numbers of CBMs have been developed and are prescribed to patients. The consequence is that results from any new pharmacokinetic studies, on single CBM interventions, cannot be accurately applied to other CBMs already exisiting on the market due to the numerous CBM compositions and administration forms. Thus, researching all CBMs is not feasible.

To date, it has also been shown that neither the dose administered nor detected plasma levels of cannabinoids are good predictors of pain alleviation when treated with CBMs. The high inter-individual variability (CV%) is likely due to patient variations in BMI, enterohepatic circulation, degradation in the gut, previous cannabinoid exposure, the endogenous cannabinoid system (endocannabinoid system) compositions and differences in administration technique[8,10]. Additionally, the subjective effects after cannabinoid administration do not appear to have a classic monophasic dose-response relationship. One such study observed that higher doses did not lead to improved effectiveness, versus lower doses, in oromucosal cannabinoid preparations (THC & CBD)[11]. Another study did however highlight dose-dependent effects on heart rate, after inhaling cannabis (THC), however this dose-dependent relationship was absent for the subjective effects associated with cannabinoid administration[12]. A recent study using vaporised cannabinoids in chronic pain patients with fibromyalgia (Bedrocan: Bediol, Bedrolite, Bedrocan & placebo) clearly showed that after single administration, the subjective changes in evoked pressure pain had a temporal relationship with plasma cannabinoid levels after administration – however only single doses were used in this study[13]. Therefore, the current best-practice is for patients administering CBMs to undergo dose-escalation titration following a premeditated dosage build-up scheme (e.g. administration 4x daily). In order to achieve an effective dose, patients continue titration until side-effects are reached, and subsequently reduce their dose by one step in the titration scheme (table 1), and subsequently maintain this dose[14]. Methods for dose-escalation vary significantly between patients in clinical practice, which is why a fixed titration scheme should be employed for research studies. This feasibly standardizes the dose-escalation for all participants in clinical studies.

Pain however is a multidimensional experience comprising physical (nociceptive) and emotional/psychological (conditioning) components. An additional challenge in EB patients is that most patients suffer from pain caused by multiple physical and pathophysiological aetiologies [15–18]. A well-known example of this, which has been described in all EB subtypes, is chronic neuropathic pain accompanied by continuous nociceptive pain from chonic wounds and inflammation of the skin or mucosa. Adding further burden is the psychological response (pain affect) to chronic pain that exacerbates and worsens one's experiences of pain. The role of nociplastic pain (pain that arises from altered nociceptive function) has not been investigated in EB, however given it's postulated role in chronic pain conditions, may indeed be relevant in EB-related pain [19]. Due to this complex nature of chronic pain in EB, adequate analgesia is not achieved by single pharmacologic interventions, and on the contrary, the side-effect burden of poly-analgesic-pharmacy outweighs the decision to prescribe multiple pharmacologic interventions.

In a recent cross-sectional study on EB in the Netherlands, patients rated 20 qualities of pain. The highest score in all subtypes in the cohort was that for an "unpleasant" quality of pain [20]. This is interesting as research has shown that pain affect, located in the limbic system, which is reported by patients as the pain quality "unpleasantness", is functionally distinct from sensory pain processing pathways. One way to measure pain affect is using functional magnetic resonance imaging (fMRI) techniques to measure brain connectivity in known and suspected areas processing pain affect [21–23]. Numerous studies using this technique have identified the amygdala and anterior cingulate cortex (ACC) as central brain areas that alter connectivity during chronic pain. Furthermore, independent of the aetiology of chronic pain, all chronic pain states (measured by fMRI) show similarities in altered brain connectivity, which are subsequently reversed (improved) with successful treatments [22].

Endogenously produced cannabinoids (endocannabinoids) play a key role in the modulation of pain signals by binding endocannabinoid receptors 1 and 2 (CB1/2) [24,25]. CB1/2 are ubiquitous, most prevalently found in the central and peripheral nervous systems, however are sparsely distributed in lower brain-stem regions for cardiovascular and respiratory functions, which explains the unattainable lethal doses and complete absence of mortality from plant derived CBMs [26–28]. As CB1/2 are densely populated in the frontal-limbic areas of the brain, their role modulating pain affect has been proposed [29]. Like these endocannabinoids, exogenous cannabinoids (CBMs), namely THC and CBD, also target these receptors; modulating neurotransmitter release in the CNS. Specifically, the amygdala and ACC are shown to have altered functional connectivity to brain cortices through CB1/2 agonism. This change in connectivity significantly correlates with a decrease in the pain, and

more specifically a decrease in the pain quality “unpleasantness” from noxious stimuli after systemic administration of Transvamix-like drugs (comprising THC and CBD) [29–31]. Generally, in clinical studies for a heterogenic group of chronic pain conditions, moderate evidence was shown for the efficacy of CBMs versus placebo [32,33]. In addition to this, in recent years, clinical studies on various painful diseases have shown the superiority of combining multiple cannabinoids compared to single-cannabinoid extracts, with specific regard to the combination of THC & CBD [34–36]. Which is also attributed to an improved tolerance of CBM interventions when both THC and CBD are combined, whereby higher doses can be administered [35,36].

1.4 Concluding Remarks

Taken together, the unmet symptomatic treatment needs in EB and moderate quality evidence for the use of Transvamix-like drugs to treat pain and modulate pain affect, open up grounds for further clinical investigation.
New fMRI techniques to assess brain connectivity will objectify both baseline characteristics in connectivity in EB patients with pain, as well as connectivity changes in areas processing pain affect. As we employ standardadized dose-escalation schemes, a pharmaceutical grade CBM intervention, validated patient reported outcomes, and a sufficient sample size, we will be able to use rigorous statistical analyses and shed light on Transvamix effects on chronic pain in EB. We hypothesize a novel avenue of pain intervention for EB with regard modulating pain affect – objectified by fMRI.

2. OBJECTIVES

2.1 Hypothesis:

- CBM-oil comprising both THC and CBD (Transvamix 10% THC / 5% CBD) can alleviate pain in patients with inherited epidermolysis bullosa.

2.2 Primary Objective

- To determine the effect of Transvamix, relative to placebo, on participant reported pain scores of the quality “unpleasantness” in EB patients with chronic pain.

2.3 Secondary Objectives

- To explore the effect of Transvamix, relative to placebo, on participant reported general pain quality scores, and pain intensity, in EB patients with chronic pain.

- To explore the effect of Transvamix, relative to placebo, on pain self-efficacy in EB patients with chronic pain.

- To explore the effect of Transvamix, relative to placebo on pruritus intensity in EB patients with chronic pain.

- To explore the effect of Transvamix, relative to placebo on brain connectivity in areas corresponding to the affective pain modulation circuit.

- To determine the sub-side effect threshold maintenance dose achieved during titration of Transvamix and placebo.

- To determine the frequency and participant-reported burden of adverse-events encountered during titration of Transvamix and placebo.

3. STUDY DESIGN

The study design is an *explorative randomized, placebo-controlled and double-blind intervention crossover study: Transvamix (100mg/mL THC / 50mg/mL CBD) to treat chronic pain in Epidermolysis Bullosa*. For further overview, see Table 1. Sixteen (16) participants will be enrolled for this study.

This is an explorative study because cannabinoid-based medicines (CBMs), Transvamix-like drugs, have not yet been studied by way of controlled trials in the EB patient population.

The addition of placebo mitigates confounding participation bias. The crossover design reduces the number of participants required, an important factor when studying rare diseases, and supported by randomization and blinding, enables rigorous statistical analyses. In addition to this, both intra-participant outcomes as well as group analyses by intervention type can be analysed.

Participants will receive both the IMP (Transvamix) and placebo in forward or reverse orders, depending on treatment arm allocation. The study drugs (Transvamix & placebo) will be provided by Felix Farma, and dispensed by the Transvaal pharmacy. A washout of two weeks between both interventions will mitigate carry-over effects as up to 80-90% of THC is excreted via the urine within 5 days (terminal elimination half life of 21.5 hours)[37], and the elimination half-life of CBD is between 1.44 and 10.86 hours after oromucosal spray administration (5–20 mg)[38]. The duration of participation is 9 weeks. All participant reported outcome measurements (PROMs) are completed online, at home.

Table 1: Table of Study-related Activities for the duration of C4EB			Until day -8	Day -7 to -1	Day 1 to 14	Day 15 to 28	Day 29 to 42	Day 55
	Time required	Location	*Eligibility Screening*	*Before intervention with Transvamix-oil or Placebo*	*Titration and Maintenance of Transvamix or Placebo*	*Washout Period (no IMP)*	*(Crossover) Titration and Maintenance of Transvamix or Placebo*	*Structured interview on study participation, events and feedback on methodology.*
Eligibility Screening; NRS pain; medication recording	60 minutes	Video conference / telephone	Completed					
Transvamix or Placebo administration	N/A	Adminstered at home			Administration following titration scheme		Administration following titration scheme	
PROM:<br>*(DN-4; CSI; HADS; PCS; SF-MPQ2; PSEQ)<br>**(MPQ2; PSEQ)	*40 minutes<br>**20 minutes	Online survey completion at home		(day -1)*	(day 14)**		(day 42)**	
Logbook (VAS Pa/Pr , dose administered, side-effects)	10 minutes	Online survey completion at home		(daily)	(daily)		(daily)	
Urine Toxicology	N/A	UMCG dermatology outpatient clinic			(day 14)		(day 29)	
fMRI scan Center for Medical Imaging UMCG)	20-30 minutes	Center for Medical Imaging UMCG		(day -1)	(day 14)		(day 42)	
Participation time in days (total = 64 days, or 9 weeks and 1 day)				7	14	14	14	14

4. STUDY POPULATION

4.1 Population (base)

Participants in this study are adult patients with the diagnosis of inherited epidermolysis bullosa (EB) and genetic diagnosis of one of the EB subtypes. All patients with EB in the Netherlands are registered at the Center for Blistering Diseases, Department of Dermatology, UMC Groningen.

In order to participate, patients must currently be living in the Netherlands and be at least 16 years of age. Participation is not limited to one sex, nor to any ethnic group. As of January 2020, 196 patients fulfil the basic demographic criteria for participation (>16 years of age, EB diagnosis, Dutch speaking and living in the Netherlands).

4.2 Inclusion criteria

In order to be eligible to participate in this study, a subject must meet all of the following criteria:

- Clinical diagnosis, supplemented by genetic analysis, immunofluorescent diagnostics or electron microscopy of congenital epidermolysis bullosa (EB). Including the subtypes *recessive dystrophic EB, dominant dystrophic EB, junctional EB* and *EB simplex.*
- At least 16 years of age from the date of onset of participation.
- Can read and write in the Dutch language.
- Mentally competent and legally able to appreciate informed consent.
- Reporting an average pain or pruritus mean score ≥4 on NRS (0-10) averaged throughout the previous week at one of the following times of day: morning, afternoon or evening.
- Negative COVID-19 testing will be required prior to participation

4.3 Exclusion criteria

A potential subject who meets any of the following criteria will be excluded from participation in this study:

- Patients enrolled in other clinical trials that do not allow for a deviation in treatment.
- Have experienced myocardial infarction or clinically significant cardiac dysfunction within the last 12 months or have had a cardiac disorder that, in the opinion of the investigator would have put the participant at risk of a clinically significant arrhythmia or myocardial infarction.

- Patients with known psychotic disorder (including the use of antipsychotic medications), or a history of suicidal ideation.
- Female patients of child-bearing potential and male participants whose partner was of child-bearing potential, unless willing to ensure that they or their partner used effective contraception.
- Patients who have had significantly impaired renal or hepatic function in the last 12 months.
- The patient is currently using or has used cannabis or cannabinoid-based medications within 30 days of study entry and was unwilling to abstain for the duration of the study.
- Patients unwilling or unable to refrain from driving road vehicles and/or using potentially dangerous machinery where sufficient concentration is necessary.
- Patients unable to stay within the Netherlands for the duration of the study period.
- History of addiction and/or hospital admission due to addiction to recreational or pharmaceutical drugs.
- Patients with contradictions for MRI determined through the MRI safety form (see *K6_Veiligheidscontrole Proefpersonen*)

4.4 Sample size calculation

We performed a sample size calculation based on a recent study ("Meds4EB" *Schräder NHB 2020*: unpublished) of pain scores measured by NRS (mean ± SD) pain score in 28 patients with pain >3 on NRS: **5.76 ± 1.45**).

1. To establish a significant reduction in pain scores of 30%, with a power of 80% and α 0.05, the minimum sample size is 9 (or 5 participants in each study arm)
2. To establish a significant reduction in pain scores of 50%, the minimum sample size is 5 (or 3 participants in each study arm).

Owing to the rarity of EB and the strict inclusion criteria for age and chronic pain, the estimated feasible participant recruitment number is 16. For this study we aim to include 16 participants (8 in each study arm).

5. TREATMENT OF SUBJECTS

5.1 Investigational product/treatment

5.1.1 Transvamix

The investigational medicinal product (IMP) is Transvamix, a cannabinoid-based medicine (CBM) comprising 100mg/mL THC (*delta-9-tetrahydrocannabinol*) and 50mg/mL CBD (*cannabidiol*) (*Transvamix-oil THC 10% / CBD 5%, 10ml*). Transvamix is prepared by Felix Farma B.V. using pharmaceutical-grade cannabis produced by Bedrocan B.V., and isolated using an ethanol-based extraction process. The end product, Transvamix, has a standardized cannabinoid composition within regulatory margins. Transvamix is administered using a 1ml syringe. (See *D2_IMPD).*

5.1.2 Placebo

The placebo formulation is comparable in texture, colour and taste. (see D2_IMPD section 3.1)

5.2 Escape medication

Participants will continue analgesic and antipruritic treatments according to their current prescriptions and epidermolysis bullosa breakthrough pain guidelines [16] – which include instructions for breakthrough symptoms. The IMP is not intended for breakthrough symptoms as participants will adhere to a strict dosing regimen. During the baseline visit participants will report all currently used systemic treatments. Participants will report changes in dosing or additional administration to these medications (for example for breakthrough medication) in the participant’s logbook.

6. INVESTIGATIONAL PRODUCT

6.1 Name and description of investigational product(s)

Transvamix THC 10% [100mg/mL] / CBD 5,0% [50mg/mL], 10 ml dark brown glass bottle with 1mL dosing syringe. (For more information see *D1_Investigators Brochure)*

6.2 Summary of findings from non-clinical studies

Please see *D1_Investigators Brochure* Chapter 4 (Nonclinical pharmacology and toxicology data)

6.3 Summary of findings from clinical studies

Please see *D1_Investigators Brochure* Chapter 5 (Effects in humans)

6.4 Summary of known and potential risks and benefits

Sublingually administered Transvamix-like drugs comprising THC and CBD are generally well tolerated. The most common treatment related adverse events are dizziness, nausea and fatigue.

Please see *D1_Investigators Brochure* Chapter 5.2 (Safety and Efficacy).

6.5 Description and justification of route of administration and dosage

The choice of administration form for this study is via the sublingual oro-mucosal route. The lipid soluble cannabinoids administered in the form of an oil are readily absorbed through the mucosa into sublingual vessels. Pharmacokinetic studies have demonstrated the fast-onset plasma concentrations and ease-of-dosing of sublingually administered cannabinoids (tetrahydrocannabinol-THC & cannabidiol-CBD), compared to oral and intrapulmonary administration forms [39–41].

Although intrapulmonary administration (for example by way of vaporization) leads to faster peak plasma concentrations, the therapeutic effects are short-lived and the risk for side-effects is higher. Through the oral route (tablet/swallowed), due to it’s poor bio-availability and the hepatic first-pass metabolism, dosing is unpredictable with a higher inter-individual variability compared to sublingual and inhaled administration routes [40,41].

The current standard in both the clinical setting and research studies point to the need for step-wise dose titrations in patients receiving CBMs comprising THC and CBD. Patients administering Transvamix and Transvamix-like drugs, by way of the

sublingual/oromucosal route, perform a dose-escalation, following a premeditated titration scheme [14,42]. The titration schemes used in clinical practice and research studies show variation in dose-escalation, the consensus however is to start with administration of the lowest dose possible, and escalate up to a maximum of 75mg THC per day, during 14 days. The scheme used in this study is designed by Felix Farma based on current recommendations for sublingually administered Transvamix-like drugs in the Netherlands.

In clinical practice this scheme this used as a reference for patients who may deviate from said doses, however for this study participants will adhere to the same dose-escalation scheme.

Taken together, the oromucosal route of administration appears to be the most patient-friendly which is supported by a recent Dutch study showing an epidemiologic preference for sublingual administration of CBMs versus oral and intrapulmonary [43].

6.6 Dosages, dosage modifications and method of administration

Transvamix and placebo are administered sublingually using a 1 millilitre dosing syringe. All participants will follow the predetermined titration scheme (*Table 2*). The titration will be stopped at the onset of side-effects (as reported by the participant), and the dose administered reduced by one day in the titration scheme. This will be called the sub-side effect threshold maintenance dose.

This achieved dose will be maintained for the remaining intervention period until day 14 is completed for each intervention (Transvamix and placebo). Both placebo and IMP (Transvamix) will undergo the same titration scheme.
The maximum dose is 0.75ml/day Transvamix or placebo (divided between 4 separate administrations).

For additional information please see *D1_Investigators Brochure* chapter 5.3.1.

**Table 2:** Titration Scheme<br>**Transvamix** (100mg/*mL* THC & 50mg/*mL* CBD) & **Placebo**					Total (ml)	Total THC (mg)	Total CBD (mg)
Day	6AM – 9AM	11AM-2PM	4PM – 7PM	9PM – 12AM			
1	-	-	-	0,05 ml	0,05 ml	5	2,5
2	-	-	-	0,05 ml	0,05 ml	5	2,5
3	0,05 ml	-	-	0,05 ml	0,1 ml	10	5
4	0,05 ml	-	0,05 ml	0,05 ml	0,15 ml	15	7,5

5	0,05 ml	0,05 ml	0,05 ml	0,05 ml	0,2 ml	20	10
6	0,05 ml	0,05 ml	0,05 ml	0,1 ml	0,25 ml	25	12,5
7	0,1 ml	0,05 ml	0,05 ml	0,1 ml	0,3 ml	30	15
8	0,1 ml	0,05 ml	0,1 ml	0,1 ml	0,35 ml	35	17,5
9	0,1 ml	0,1 ml	0,1 ml	0,1 ml	0,4 ml	40	20
10	0,1 ml	0,1 ml	0,1 ml	0,15 ml	0,45 ml	45	22,5
11	0,15 ml	0,1 ml	0,1 ml	0,15 ml	0,5 ml	50	25
12	0,15 ml	0,1 ml	0,15 ml	0,15 ml	0,55 ml	55	27,5
13	0,15 ml	0,15 ml	0,15 ml	0,2 ml	0,65 ml	65	32,5
14	0,2 ml	0,15 ml	0,2 ml	0,2 ml	0,75 ml	75	37,5

6.7 Preparation and labelling of Investigational Medicinal Product

Production of cannabis flos (the whole dried flower) is strictly regulated by the Dutch government. The task for this regulation is seeded with the Office of Medicinal Cannabis (Bureau voor Medicinale Cannabis (BMC, part of Ministerie van VWS)). The raw material (cannabis flos) is produced by Bedrocan BV, Veendam NL (GMP with a constant composition of cannabinoids and terpenes.

Bedrocan produces standardized Active Pharmaceutical Ingredient (API). These products are used by the pharmaceutical industry, and meet the needs of large and small drug developers. This is because every stage of the manufacturing process is GMP-certified. Bedrocan is the world’s only GMP producer of standardized, full-flower, medicinal cannabis. It offers both blended and genetically distinct herbal preparations suitable to develop novel, cannabis-based medicines. Bedrocan produces standardized cannabis flos for the Office for Medicinal Cannabis (OMC), the government office with a monopoly on medicinal cannabis supply to Dutch pharmacies, and on its import and export. The OMC is also responsible for overseeing the production of cannabis for medicinal and scientific purposes, guaranteeing quality through supervision of the grower and distributor.

The Transvaal Pharmacy in The Hague was the first pharmacy to produce cannabis oil after the OMC asked them to make formulation suitable for children. Cannabis oil is comprised of a concentrated soft extract obtained by extraction of the dried flowers of the cannabis plant and pharmaceutical grade vegetable oil. The purpose of producing cannabis oil is to make cannabinoids and other beneficial components, such as terpenes, available in a highly concentrated form and to provide an alternative route of administration (other than smoking, vaporization or orally).

In the Netherlands, pharmacies can’t apply for a manufacturers authorization. For this reason, Felix Farma, which is a related party of Transvaal Pharmacy, has applied for this authorization. Currently, authorization is granted. A manufacturer authorisation number will soon be created by Farmatec.

Felix Farma will produce and label the cannabis oil. Transvaal Pharmacy will distribute the oil to the patients.
For more details see *D5_Productgegevens apothekers* and *D3_Voorbeeldetiketten*.

6.8 Drug accountability

Full drug accountability will be performed by Felix Farma B.V. In accordance with good clinical practice (GCP) guidelines. For more details see *D5_Productgegevens apothekers*.

7. NON-INVESTIGATIONAL PRODUCT

Not applicable

8. METHODS

8.1 Study parameters/endpoints

8.1.1 Main study parameter/endpoint

- A reduction of 30% and 50% of mean pain scores (of the pain quality “unpleasantness”) between Transvamix and placebo group.
  Will be measured as the difference in mean scores pre- (baseline) and post-intervention (Transvamix and placebo) for each group using the pain quality items for “unpleasantness” from the short-form McGill Pain Questionairre 2 (SF-MPQ2), a 22-item numerical rating scale (NRS 0-10) survey on pain qualities.

8.1.2 Secondary study parameters/endpoints.

- The difference of mean scores of pain qualities between Transvamix and placebo.
  Will be measured as the difference of the mean of all SF-MPQ2 pain quality items (which have received a score >0 on NRS) between Transvamix and Placebo.
- The difference of mean scores of pain intensity between Transvamix and placebo.
  Will be measured as daily changes in pain intensity (1-14 days placebo, 1-14 days Transvamix) using the VAS-Pain ([no pain] 0-100 [worst pain imaginable] unit scale) for pain intensity. The change score (delta) from baseline to Transvamix (day 14) and Placebo (day 14) will be calculated.
- To determine the effect of Transvamix on pain self-efficacy
  Will be measured using the PSEQ survey pre-intervention (1x baseline) and during intervention (1x Transvamix, 1x placebo), a 10-item survey on the ability to continue normal life given ongoing pain.
- The difference of mean scores of pruritus intensity between Transvamix and placebo.
  Will be measured as daily changes in pruritus intensity (1-14 days placebo, 1-14 days Transvamix) using the VAS-Pruritus ([no pruritus] 0-100 [worst pruritus imaginable] unit scale) for pruritus intensity. The change score (delta) from baseline to Transvamix (day 14) and Placebo (day 14) will be calculated.
- The difference in brain connectivity of the seed regions (anterior cingulate cortex and amygdala) to the brain cortex between Transvamix and placebo.
  Will be measured using voxel-based analysis of blood oxygen-level dependent functional magnetic resonance imaging (fMRI-BOLD).

- To determine sub-side effect threshold maintenance dose achieved of Transvamix during titration and the respective interindividual variability (CV%). *Will be measured as the dose administered on day 14 of placebo and Transvamix interventions. CV% is measured as inter-participant variance of dose achieved on day 14 of placebo and Transvamix interventions.*
- To inventorize how many and which participant-experienced adverse-events during titration and maintenance of Transvamix and the patient reported burden for each adverse event on numeric rating scale (NRS [not burdensome at all] 0-10 [extremely burdensome]).

8.2 Randomisation, blinding and treatment allocation

Blinding and the master file to blinded data will be managed by Felix Farma B.V. Randomization to each treatment arm will be completed by Felix Farma B.V. (see *D5_Productgegevens Apothekers*).

8.3 Study procedures

After meeting all the inclusion criteria and enrolling participants in this study, all reported outcomes including both research clinician and participant reported survey will be recorded in the online case report forms (CRF) built into the traceable research program RedCap. The clinical research office at the UMCG will ensure the PI, research clinician and monitors have access to the CRFs and outcome measurement data in RedCap. Participants will receive URLs to access the surveys for online completion from a smartphone, tablet or computer.

8.3.1 Daily participant logbook completed by participants.

Participants will complete a logbook consisting of 3 parts each day in the evening (table 1) throughout the study period during which the participant received either Transvamix or placebo:

- Visual analog scales for pain and pruritus (VAS [no pain] 0-100 [worst pain/pruritus imaginable] units). These will be completed in the evening. Participants will be asked to complete VAS pain/pruritus retrospectively about the same day, subdivided into 3 timepoints in the day (*morning*, *afternoon* and *evening* [or *now*]).

  The periods of completion are as follows: Firstly, for baseline measurements (day -8 to -1) followed by Transvamix and placebo interventions (days 1 - 14 and 29 – 42).

- The titration scheme will be visualised and a checkbox ticked by the participant indicating the day of titration that was completed.
- Side effects will be reported following a multiple choice list*, and the burden of the noted side effect indicated using numeric rating scales (NRS [not burdensome] 0-10 [extremely burdensome])
- Use of breakthrough medication or changes in analgesic medication recorded by participant.

  * Multiple choice list of side effects are categorized (in Dutch):
  1. High gevoel (een vrolijke opgewekte stemming die langzaam overgaat in een tevreden gevoel van kalmte en rust).
  2. Loomheid
  3. Honger
  4. Intensere beleving van kleuren en/of geluiden
  5. Verlies van gevoel voor tijd en/of plaats
  6. Sombere en/of angstige stemming
  7. Rusteloosheid en/of slapeloosheid
  8. Waanvoorstellingen
  9. Versnelde hartslag
  10. Duizeligheid
  11. Warm of koud gevoel in handen en voeten
  12. Rode branderige ogen
  13. Spierverslapping
  14. Droge mond
  15. Anders, namelijk.....

8.3.2 Surveys completed by participants

The short form McGill Pain Questionnaire (SF-MPQ2) and pain self-efficacy questionnaires (PSEQ) will be completed 3 times. Firstly at baseline (day -1), followed by the last day of both Transvamix and placebo interventions (days 14 and 42).

The douleur neuropathique 4 (DN-4), central sensitisation index (CSI), hospital anxiety and depression survey (HADS) and pain catastrophizing scale (PCS) are additional surveys that will be completed at baseline (day -1) only.

8.3.3 Functional Magnetic Resonance Imaging

During eligibility screening for the study, the MRI safety form will be completed and checked by the research clinician.

The fMRI scans will be repeated on three occasions at the cognitive neuroscience centre, UMCG. Firstly, the baseline will be obtained prior to intervention (pre-dose measurement). The second fMRI scan will take place on day 14. The third fMRI

scan on day 42.

Each scanning procedure consists of two parts:

1. T1 weighted anatomical scan (10 minutes)
2. Resting state scan (10 minutes – participants are instructed to close eyes, not fall asleep and let their minds roam freely. Heart rate and breathing rate will be recorded continuously).

8.3.4 Urine THC-COOH Toxicology

Urine samples will be collected and analyzed at the department of clincal toxicology UMCG. During the study, urine toxicology will be assessed for THC-COOH at 2 time points: On day 14 and 42 (on the last day of Transvamix and placebo intervention). Participants will collect first-urine in the morning (before morning administration) and take this to the study clincal visit, to be collected by the research clinician. Toxicology results will not be revealed until the end of the study period, after the data analysis for the primary outcome is complete.

8.3.5 Follow-up Structured Interview

Participants who have completed participation, or who have dropped out (and have given consent for regular follow-up) will be contacted by telephone or video conferencing by the research clinician. The interview will consist of 3 parts:

1. General perspectives on participation (related to recruitment and study design)
2. General perspectives on interventions (related to Transvamix and placebo).
3. General and specific feedback for future studies pertaining to CBMs as interventions and/or EB patients.

Directly following the structured interview, participants will be sent via email an automated researcher-blinded question on the order of interventions received (the responses will be unblinded after statistical analysis of the study is completed). The options are: I) Transvamix → Placebo, II) Placebo → Transvamix, III) I don't know.

8.4 Withdrawal of individual subjects

Subjects can leave the study at any time for any reason if they wish to do so without any consequences. The investigator can decide to withdraw a subject from the study for urgent medical reasons.

8.5 Replacement of individual subjects after withdrawal

In case of participant or research physician decided withdrawal, the recruitment will continue until n=16 have been enrolled and completed participation in the study.

8.6 Follow-up of subjects withdrawn from treatment

The researchers will ask the subject who has withdrawn to clarify whether they wish to withdraw from all components of the trial or allow for the standard follow-up to take place at 14 days post end of study (which was consented to when commencing participation).

8.7 Premature termination of the study

The study can be terminated by the investigators in case of:

- Inability to enrol adequate numbers of participants
- Financial restraints
- Problems with the manufacturing or stability of the study medication
- Failure of researchers to follow good clinical practice standards
- Unacceptable changes in personnel / researchers / investigators.

9. SAFETY REPORTING

9.1 Temporary halt for reasons of subject safety

In accordance to section 10, subsection 4, of the WMO, the sponsor will suspend the study if there is sufficient ground that continuation of the study will jeopardise subject health or safety. The sponsor will notify the accredited METC without undue delay of a temporary halt including the reason for such an action. The study will be suspended pending a further positive decision by the accredited METC. The investigator will take care that all subjects are kept informed.

9.2 AEs, SAEs and SUSARs

9.2.1 Adverse events (AEs)

Adverse events are defined as any undesirable experience occurring to a subject during the study, whether or not considered related to Transvamix or placebo. All adverse events reported spontaneously by the subject or observed by the investigator or his staff will be recorded.

9.2.2 Serious adverse events (SAEs)

A serious adverse event is any untoward medical occurrence or effect that

- results in death;
- is life threatening (at the time of the event);
- requires hospitalisation or prolongation of existing inpatients' hospitalisation;
- results in persistent or significant disability or incapacity;
- is a congenital anomaly or birth defect; or
- any other important medical event that did not result in any of the outcomes listed above due to medical or surgical intervention but could have been based upon appropriate judgement by the investigator.

An elective hospital admission will not be considered as a serious adverse event.

The investigator will report all SAEs to the sponsor without undue delay after obtaining knowledge of the events.

The sponsor will report the SAEs through the web portal *ToetsingOnline* to the accredited METC that approved the protocol, within 7 days of first knowledge for

SAEs that result in death or are life threatening followed by a period of maximum of 8 days to complete the initial preliminary report. All other SAEs will be reported within a period of maximum 15 days after the sponsor has first knowledge of the serious adverse events.

9.2.3 Suspected unexpected serious adverse reactions (SUSARs)

Adverse reactions are all untoward and unintended responses to an investigational product related to any dose administered.

Unexpected adverse reactions are SUSARs if the following three conditions are met:

1. the event must be serious (see chapter 9.2.2);
2. there must be a certain degree of probability that the event is a harmful and an undesirable reaction to the medicinal product under investigation, regardless of the administered dose;
3. the adverse reaction must be unexpected, that is to say, the nature and severity of the adverse reaction are not in agreement with the product information as recorded in:
   - Summary of Product Characteristics (SPC) for an authorised medicinal product;
   - Investigator's Brochure for an unauthorised medicinal product.

The sponsor will report expedited the following SUSARs through the web portal *ToetsingOnline* to the METC:

- SUSARs that have arisen in the clinical trial that was assessed by the METC;
- SUSARs that have arisen in other clinical trials of the same sponsor and with the same medicinal product, and that could have consequences for the safety of the subjects involved in the clinical trial that was assessed by the METC.

The remaining SUSARs are recorded in an overview list (line-listing) that will be submitted once every half year to the METC. This line-listing provides an overview of all SUSARs from the study medicine, accompanied by a brief report highlighting the main points of concern.

The expedited reporting of SUSARs through the web portal Eudravigilance or ToetsingOnline is sufficient as notification to the competent authority.

The sponsor will report expedited all SUSARs to the competent authorities in other Member States, according to the requirements of the Member States.

The expedited reporting will occur not later than 15 days after the sponsor has first knowledge of the adverse reactions. For fatal or life threatening cases the term will be maximal 7 days for a preliminary report with another 8 days for completion of the report.

9.3 Annual safety report

In addition to the expedited reporting of SUSARs, the sponsor will submit, once a year throughout the clinical trial, a safety report to the accredited METC, competent authority, and competent authorities of the concerned Member States.
This safety report consists of:

- a list of all suspected (unexpected or expected) serious adverse reactions, along with an aggregated summary table of all reported serious adverse reactions, ordered by organ system, per study;
- a report concerning the safety of the subjects, consisting of a complete safety analysis and an evaluation of the balance between the efficacy and the harmfulness of the medicine under investigation.

9.4 Follow-up of adverse events

All AEs will be followed until they have abated, or until a stable situation has been reached. Depending on the event, follow up may require additional tests or medical procedures as indicated, and/or referral to the general physician or a medical specialist. SAEs need to be reported till end of study within the Netherlands, as defined in the protocol

9.5 Data Safety Monitoring Board (DSMB) / Safety Committee

Using the NFU risk classification table and recommendations it has been determined that the study risk for participants is negligible. However, due to the nature of Transvamix as a compounded medication which is readily prescribed, whereby basic data on safety and tolerability is unknown, a DSMB will be set-up to monitor preliminary study data pertaining to adverse events throughout the study period.
Additional information on the template can be found in *K5_DSMB Charter Template.*

10. STATISTICAL ANALYSIS

10.1 Primary study parameter(s)

The NRS values for the SF-MPQ2 items “unpleasantness” (also referred to as negative pain affect) will be assessed as the primary outcome.
The data will be presented using descriptive statistics (means and standard deviations, or median and interquartile ranges), and subdivided into three groups (baseline, placebo and Transvamix). The change scores for baseline to placebo and Transvamix will be computed and incorporated for statistical testing. Shapiro Wilk will be used to check for normality, and either t-testing (paired and independent samples t-testing) or non-parametric tests (including wilcoxin) will be used depending on the distribution observed. The significance level will be set to $p<0.05$. In addition, mixed effects models will be applied, allowing for analysis of longitudinal data, missing data and possible period effects.

10.2 Secondary study parameter(s)

10.2.1 Participant Reported Outcome Measurements (PROMs)

For all PROMs (SF-MPQ2 [all items], PSEQ [all items] and VAS pain/pruritus), descriptive statistics will be employed to portray means and standard deviations or medians and interquartile ranges for the measurements: baseline, placebo and Transvamix. The number of adverse events, and the participant reported burden of each, will be presented for each group (placebo and Transvamix) using frequencies of reported adverse events, means with standard deviations or median with interquartile ranges (depending observed distribution).

10.2.2 Dose achieved

Descriptive statistics will be employed to portray means, medians, coefficient of variation (CV%) and standard deviations of achieved doses for the entire cohort. No sub-group analyses will be performed on dose-achieved. The dose achieved, is the dose administered by the participant during the sub-side effect threshold maintenance period – as seen on day 14 and 42 of the study.

10.2.3 fMRI analysis

All anatomical regions of interest (ROI) will be defined using the Harvard Oxford Cortical and Subcortical Structural Atlas (http://www.fmrib.ox.ac.uk/fsl/ data/atlas-descriptions.html), which is a probabilistic population- based atlas. Only voxels estimated at >50% of probability of being in the structure are included in the ROI. Anatomical areas of interest have known association with affective pain-related pathways.

The effects of Transvamix will be explored by measuring the functional connectivity between the seed regions, the anterior cingulate cortex (ACC) and

amygdala, to other areas of interest in the brain by using BOLD fMRI resting state data. The intra-individual (per participant) effect-size will be analysed by comparing changes in brain connectivity (baseline vs. Transvamix vs. Placebo). On a group level the effects of the intervention will be explored using classical mass-univariate analysis and correlations against, for example, maintenance dose and VAS-Pa/Pr. Furthermore, appropriate machine learning techniques will be used to reveal co-occurring networks of brain connectivity changes. Heart rate and respiratory rate will be recorded and used as a confound if needed.

Data will be represented using descriptive statistics (mean, standard deviation or median and inter-quartile ranges). Statistical testing against the null hypothesis will be performed using non-parametric, permutation analysis.

10.3 Other study parameters

10.3.1 Baseline measurements

The DN-4, CSI, PCS and HADS are completed at baseline only. These data will be incorporated retrospectively in post-hoc data analysis, whereby responders can be grouped according to cutoff scores for plausible neuropathic pain (DN-4), plausible central sensitisation (CSI), having pain catastrophizing traits (PCS) and having characteristics of anxiety and depression (HADS), as descriptors for underlying EB-related pain etiology.

10.3.2 Follow-up Structured Interview

The follow-up interview will be recorded and transcribed into an online format. A 2-step thematic analysis by two members of the research team (to allow for cross-checking/verification) will be used to analyse the responses. The first step identifies codes the transcribed participant responses, and the second step reviews the codes and categorized them into themes. A list of themes is the product of the qualitative analysis.

The participant’s response to the order of interventions given will be assessed as after all other data analysis has been completed. The number of participants correctly/incorrectly guessing the order of interventions will be presented as percentages – consequences of this data will be revealed in the study discussion.

10.4 Interim analysis (if applicable)

An interim analysis is not planned for this study.

11. ETHICAL CONSIDERATIONS

11.1 Regulation statement

This research will be conducted according to the principles of the Declaration of Helsinki (amended by 64th WMA General Assembly, Fortaleza, Brazil, October 2013), version 9th July 2018. This study will be performed in accordance with the Medical Research Involving Human Subjects Act (WMO). The procedures set out in this study protocol are designed to ensure that the Sponsor and investigator abide by the Guidelines of the Good Clinical Practice (GCP) of the European Community (EC) (ICH topic E6, CPMP/ICH/135/95, Directive 2001/20/EC) and the Declaration of Helsinki (in the latest revised version) in the conduct, evaluation and documentation of this study.

11.2 Recruitment and consent

Potential participants in this study are all registered at the Center for Blistering Diseases, Department of Dermatology, University Medical Center of Groningen. The Center for Blistering Diseases is the tertiary referral centre for all patients with *epidermolysis bullosa* in the Netherlands with regard to treatment, follow-up and (non-) clinical research with patients.

Potential participants, with known EB diagnosis, and who visit the Center for Blistering Diseases for regular follow-up will be informed about the study. Potential participants will receive the patient information letter about the study as well as the informed consent, to take home to read, and asked to respond within 1 month about willingness to participate. They will receive contact details of the researcher and independent physician/expert. Preliminary questions from potential participants will be answered by/discussed with the researcher through a telephone call, video conference, or in person at the outpatient of the department of dermatology, UMC Groningen. If willing to participate, and the informed consent has been signed by both the participant and researcher, the researcher will liaison the screening (pertaining to inclusion and exclusion criteria) for participants.

11.3 Objection by minors or incapacitated subjects (if applicable)

Not applicable

11.4 Benefits and risks assessment, group relatedness

The use of *cannabinoid-based medicines* (CBM) has been increasing on both a Dutch national and international level. This includes an increasing number of patients with epidermolysis bullosa who are treated with CBMs for pain and pruritus with the subsequent goal to improve quality of life. The use of CBMs is largely unregulated, and clinician supervision is sparse. As CBM treatments are generally not financially

reimbursed by Dutch insurance companies, patients who look for this treatment outside a regulated healthcare setting cannot be supervised by clinicians.

Recently EB clinicians in the Netherlands and around the world have been prescribing CBMs with largely positive responses from patients. Objective evidence for the effectiveness of this treatment has yet to be established, and thus clinicians have no evidence or guidelines to work with. This is disappointing for both patients and clinicians. As the CBM formulation and administration form for this study has been used as a prescription drug in the Netherlands, with a high tolerance level and a reportedly safe profile, it seems necessary as the following step to administer this treatment intervention in the form of a clinical study, and report the relative effects versus placebo. Patients with moderate-severe EB in the Netherlands, are continuously discussing new treatments and proposing changes to their clinicians, because of severe symptoms. This is simply because current symptomatic treatments for EB, like a number of other painful and itchy diseases, is inadequate. In this study patients will not be required to stop current medications and therefore are not expected to have worsening of symptoms as compared with care as usual.

Patients with EB understand that their disease is not curable, however they continuously, in active cooperation with clinicians, seek treatment alternatives which is reflected by empirically-based prescribing, and ongoing clinical research studies. There is no financial compensation, other than reimbursing travel and lodging costs, for patients participating in this study, nor any penalties for either withdrawing from the study (at any time point) or refusing participation.

We hope to share our results with the worldwide EB patient and clinician community in order to further understand the effectiveness of CBMs in EB patients with chronic pain.

11.5 Compensation for injury

The sponsor/investigator has a liability insurance which is in accordance with article 7 of the WMO.

11.6 Incentives

Exrta costs made for travelling to and from the UMCG will be reimbursed.

ADMINISTRATIVE ASPECTS, MONITORING AND PUBLICATION

12. Handling and storage of data and documents

Data will be handled with confidentiality. A subject identification code list will be used to link the data to the subject. The code will not be based on the patient initials and birth date. The key to the code will be safeguarded by the principal investigator.

Data will be accessible for the team of investigators, METC and the *Inspectie voor de Gezondheidszorg*.

Data will be saved for 15 years. Data will/can be used for publication, but no subject will be traceable. The handling of personal data will comply with the Dutch Personal Data Protection Act

12.1 Monitoring and Quality Assurance

The monitoring is carried out by those responsible for this trial. The research office monitors the trial on a regular basis. This typically includes the following checks:

- The data collected are consistent with adherence to the trial protocol
- CRFs are completed by authorized persons
- No key data are missing
- The data appear to be valid (i.e. range and consistency checks)
- Review of recruitment rates, withdrawals and losses to follow-up

12.2 Amendments

Amendments are changes made to the research after a favourable opinion by the accredited METC has been given. All amendments will be notified to the METC that gave a favourable opinion.

A ‘substantial amendment’ is defined as an amendment to the terms of the METC application, or to the protocol or any other supporting documentation, that is likely to affect to a significant degree:

- the safety or physical or mental integrity of the subjects of the trial;
- the scientific value of the trial;
- the conduct or management of the trial; or
- the quality or safety of any intervention used in the trial.

All substantial amendments will be notified to the METC and to the competent authority.

Non-substantial amendments will not be notified to the accredited METC and the competent authority, but will be recorded and filed by the sponsor.

12.3 Annual progress report

The sponsor/investigator will submit a summary of the progress of the trial to the accredited METC once a year. Information will be provided on the date of inclusion of the first subject, numbers of subjects included and numbers of subjects that have completed the trial, serious adverse events/ serious adverse reactions, other problems, and amendments.

12.4 Temporary halt and (prematurely) end of study report

The sponsor will notify the accredited METC and the competent authority of the end of the study within a period of 90 days. The end of the study is defined as the last patient’s last visit.

The sponsor will notify the METC immediately of a temporary halt of the study, including the reason of such an action.

In case the study is ended prematurely, the sponsor will notify the accredited METC and the competent authority within 15 days, including the reasons for the premature termination.

Within one year after the end of the study, the investigator/sponsor will submit a final study report with the results of the study, including any publications/abstracts of the study, to the accredited METC and the Competent Authority.

12.5 Public disclosure and publication policy

This study will be registered in a public trial registry before the first patient is recruited. Results of this project will be disclosed unreservedly.

13. STRUCTURED RISK ANALYSIS

13.1 Potential issues of concern

a. Level of knowledge about mechanism of action

THC and CBD are both Cannbinoid-binding receptor (CB1/2) agonists. These receptors are distributed in the human central nervous system (excluding essential autonomous areas for breathing and heart contractility in the brainstem). These receptors are a significant part of the human endocannabinoids system (ECS) comprising enodcannabinoid receptor agonists, CB1/2 and cannabinoid-like receptors, synthesis and degradation enzymes and cellular transporters. The postulated mechanism on modulating chronic pain or pruritus is through depolarisation induced suppression of inhibition or activation in neural synapses in both the central nervous system and periphery [44]. Cannabinoid receptor agonists are able to modulate nociceptive thresholds by regulating neuronal activity, but they also modulate pain sensation by acting on non-nervous tissues [45]. The central acting cannabinoids have been shown to modulate amygdala and ACC functional connectivity leading to an altered processing of pain affect.

b. Previous exposure of human beings with the test product(s) and/or products with a similar biological mechanism.

Numerous controlled studies have assessed the safety, tolerability and effectiveness of CBMs. Patients with pain from varying aetiologies have been the largest group studied. Recent meta-analyses show results that portray a high safety and tolerability of CBM interventions [46]. There is moderate quality evidence that CBMs relative to placebo might mitigate subjective pain reporting in patients with chronic pain. There is enough evidence on the effectiveness of CBM preparations to warrant further clinical trials in numerous chronic pain conditions [33,47,48].

There have been no clinical trials to date studying the efficacy of CBMs (comprising THC or CBD) in patients suffering from *epidermolysis bullosa* (EB). However, two case-series have been published. Effects reported were reduced pain and pruritus, weaning of central acting analgesics and improved wound healing. Patients were either completely weaned off pain medication (including opioids) or had a substantial dose reduction [2,49].

c. Can the primary or secondary mechanism be induced in animals and/or in *ex-vivo* human cell material?

Not applicable.

d. Selectivity of the mechanism to target tissue in animals and/or human beings

Not applicable

e. Analysis of potential effect

The current standard for dosing CBMs is through a titration period where patients titrate the medication to the required effect – in clinical practice, which is mirrored in this study, we refer to this window as the sub-side effect threshold maintenance dose. This method is used worldwide for most CBM medications and is currently supported by the Bureau Medicinale Cannabis in the Netherlands (*Ministerie VWS*) where dosing is slowly increased to match the symptomatic needs of the patient and effect of the CBM. The most comparable Transvamix-like drug, registered in the EMA and FDA employs a titration period of up to 14 days (SmPC Sativex, MA: PL 18024/0009).

f. Pharmacokinetic considerations

See *D1_Investigators Brochure* chapter 4 (Nonclinical and pharmacology and toxicology data).

g. Study population

The study population includes patients with the clinical, genetic, direct-immunofluorescent microscopy or electronmicroscopic diagnosis of any of the three major subtypes of inherited epidermolysis bullosa (EB-simplex, junctional-EB, dystrophic-EB). Patients with EB experience severe and often refractory pain due to severe disease presentation and inadequate therapies. These are patients who are receiving routine outpatient care at the UMCG. Patients must be mentally competent, from the age of 16 years old.

The pathophysiologic mechanism intervened in this study pertains to the top-down modulation of chronic pain (pain affect) which is by nature a complex symptom. This means that ex-vivo studies are not possible, and animal-model studies have a limited capacity or cannot show results that translate to pain changes in patients with EB.

h. Interaction with other products

See D1_Investigators Brochure chapter 5.2.3 (Drug-drug interactions).

i. Predictability of effect

Changes in pain can be measured using outcome instruments (VAS/SF-MPQ2/PSEQ). Additionally functional brain connectivity with from seed regions (amygdala and ACC) are objective markers of changes in pain affect.

j. Can effects be managed?

The titration scheme use by patients/participants allows for a low dose to be initiated and step-wise increased until adequate effects are reached for patients. There are no anti-

dotes required for experienced side effects from either CBD or THC in Transvamix-oil. The possible acute onset side effects, such as dizziness or an increased appetite are short lasting (1-2 hours, and completely withdrawn 4-8 hours after administration) and can be avoided by reducing the following dose administered to the previous step in the titration scheme. In case of accidental overdose, patients are advised to go to a quiet area and just wait until the adverse effects wear off. In case of emergencies patients will have access to adequate medical support.

k. Why are both THC and CBD present in the study medication?

Clinical studies have shown that the combination of THC and CBD are superior to either cannabinoid alone. Additional CBD has been shown to reduce the unwanted side effects of THC administration through receptor antagonism, making the combination more preferable to patients receiving CBM treatments [9,35,36,50].

l. Are cannabinoid-based medicines addictive?

The key component responsible for the psychotropic effects and dependence potential of cannabinoid-based drugs is THC, acting on pathways in the central nervous system. Substance abuse can have both physical and psychological influences. Dependence is preceded by an increased tolerance to the drug and negative withdrawal symtpoms, followed by psychosocial consequences.

To address the former, a review of a CBM (comprising THC and CBD) in 2011 with >1500 patient-years of treatment experience showed the absence of increased tolerance to the study drugs – i.e. the dose remained the same throughout the studies or even was reduced [51,52]. Dutch data on the number of CBM prescriptions show an unchanged duration of use (first dispensation to last dispensation) between 2003-2011 and 2012-2016, and the average daily administration remaining remarkably stable [43].

The abuse potential of CBMs comprising THC however cannot be directly compared with commercial THC preparations as the underlying motivation to administer the drug in disease is to relieve symptoms in the absence of side-effects. In addition to this the administration form used most often recreationally, with a higher risk for abuse, is intrapulmonary, with higher percentages of active THC, leading to fast onset and high plasma peaks, and an increased chance for withdrawal symptoms [53]. This is not the case in current regulated CBMs following dose-titration schemes with supervision.

Therefore by following a dose-titration scheme and minimizing the dose administered, such as in this clinical study, the symptomatic relief is maximized and unwanted side effects are minimized.

13.2 Synthesis

CBMs in the Netherlands have gained special status on the Dutch prescription pharmaceutical market and is prescribed when other registered treatments are deemed ineffective or futile. Evidence produced by Dutch local academic institutions is lacking. The production of pharmaceutical grade medical cannabis is approved by the Ministry of Health of the Netherlands, and the extraction process forming the oil composition follows GMP guidelines regulated by the government board of health inspection. Variations in the composition of cannabinoids of the CBM-oil used in this study have been readily available on prescription since 2015 and no serious or life-threatening events have been reported to the ministry of health. The study drug Transvamix (THC 100mg/mL (10%), CBD 50mg/mL (5%)), is being developed under GMP regulations for the application in clinical studies. Phase I and II have not been required for dispensation to patients in the Dutch healthcare setting.

The safety profile of this combination of cannabinoids is well understood, however this is the first time that Transvamix is being used in a clinical study on patients with epidermolysis bullosa (EB). Although this is a rare disease, the mechanisms of top-down symptoms (pain) in EB overlap other painful diseases which have been studied in the context of CBM trials. This study is designed to safely monitor patients throughout, as well as minimize burden of participation. Participants are allowed to use breakthrough and co-medications so as to not lead to additional patient suffering during participation.

In summary this explorative study will reciprocate current clinical practice in order to measure the lay a foundation for future studies in this field. The controlled study setting allows for both the CBM intervention to be supervised as well as closely monitor (possible) changes in patient outcomes, and brain connectivity. This objective data will cornerstone the directions of future clinical research in EB and CBMs.

14. REFERENCES

1 Yuen WY, Frew JW, Veerman K, *et al.* Health-related quality of life in epidermolysis bullosa: Validation of the dutch QOLEB questionnaire and assessment in the dutch population. *Acta Derm Venereol* 2014; **94**:442–7.

2 Schräder NHB, Duipmans JC, Molenbuur B, *et al.* Combined tetrahydrocannabinol and cannabidiol to treat pain in epidermolysis bullosa: a report of three cases. *Br J Dermatol* 2019; **180**:922–4.

3 Martinez AE. Time to drop the stigma: cannabinoids are drugs that may alleviate pain in people with epidermolysis bullosa. *Br J Dermatol* 2019; **180**:711–2.

4 Medicinale cannabis minder dan 50.000 keer verstrekt [WWW Document]. Sticht. Pharm. Kernget. 2019; :51–2.

5 McCartney D, Benson MJ, Suraev AS, *et al.* The effect of cannabidiol on simulated car driving performance: A randomised, double-blind, placebo-controlled, crossover, dose-ranging clinical trial protocol. *Hum Psychopharmacol* 2020; **35**. doi:10.1002/hup.2749.

6 Guy GW, Flint ME. A single centre, placebo-controlled, four period, crossover, tolerability study assessing, pharmacodynamic effects, pharmacokinetic characteristics and cognitive profiles of a single dose of three formulations of cannabis based medicine extracts (CBMEs) (GWPD9901), plus a two period tolerability study comparing pharmacodynamic effects and pharmacokinetic characteristics of a single dose of a cannabis based medicine extract given via two administration routes (GWPD9901 EXT). In: *Cannabis: From Pariah to Prescription*. , 2014.

7 Wang T, Collet J-P, Shapiro S, Ware MA. Adverse effects of medical cannabinoids: a systematic review. *CMAJ* 2008; **178**:1669–78.

8 Guy GW, Robson PJ. A phase I, double blind, three-way crossover study to assess the pharmacokinetic profile of Cannabis Based Medicine Extract (CBME) administered sublingually in variant cannabinoid ratios in normal healthy male volunteers (GWPK0215). *J Cannabis Ther* 2003. doi:10.1300/J175v03n04_02.

9 Russo EB. Cannabinoids in the management of difficult to treat pain. *Ther Clin Risk Manag* 2008; **4**:245–59.

10 Karschner EL, Darwin WD, Goodwin RS, *et al.* Plasma cannabinoid pharmacokinetics following controlled oral Δ 9-tetrahydrocannabinol and oromucosal cannabis extract administration. *Clin Chem* 2011. doi:10.1373/clinchem.2010.152439.

11 Portenoy RK, Ganae-Motan ED, Allende S, *et al.* Nabiximols for opioid-treated cancer patients with poorly-controlled chronic pain: A randomized, placebo-controlled, graded-dose trial. *J Pain* 2012; **13**. doi:10.1016/j.jpain.2012.01.003.

12 Ramesh D, Haney M, Cooper ZD. Marijuana’s dose-dependent effects in daily marijuana

smokers. *Exp Clin Psychopharmacol* 2013; **21**. doi:10.1037/a0033661.

13 Van De Donk T, Niesters M, Kowal MA, *et al.* An experimental randomized study on the analgesic effects of pharmaceutical-grade cannabis in chronic pain patients with fibromyalgia. *Pain* 2019; **160**. doi:10.1097/j.pain.0000000000001464.

14 Geneesmiddelen Informatieblad - Bijsluiter Cannabis Olie Transvaal Apotheek. , 2019URL https://www.transvaalapotheek.nl/wp-content/uploads/2018/05/BIJSL-Cannabis-olie-19.pdf.

15 Mellerio JE, El Hachem M, Bellon N, *et al.* Emergency management in epidermolysis bullosa: Consensus clinical recommendations from the European reference network for rare skin diseases. Orphanet J. Rare Dis. 2020. doi:10.1186/s13023-020-01403-x.

16 Goldschneider KR, Good J, Harrop E, *et al.* Pain care for patients with epidermolysis bullosa: best care practice guidelines. *BMC Med* 2014; **12**:178.

17 Brun J, Chiaverini C, Devos C, *et al.* Pain and quality of life evaluation in patients with localized epidermolysis bullosa simplex. *Orphanet J Rare Dis* 2017; **12**:119.

18 Fortuna G, Aria M, Cepeda-Valdes R, *et al.* Pain in patients with dystrophic epidermolysis bullosa: Association with anxiety and depression. *Psychiatry Investig* 2017. doi:10.4306/pi.2017.14.6.746.

19 Aydede M, Shriver A. Recently introduced definition of ‘nociplastic pain’ by the International Association for the Study of Pain needs better formulation. Pain. 2018. doi:10.1097/j.pain.0000000000001184.

20 Schräder N, Yuen W, Jonkman M. Pain Quality Assessment Scale for Epidermolysis Bullosa. *Acta Derm Venereol* 2017. doi:10.2340/00015555-2827.

21 Morton DL, Sandhu JS, Jones AKP. Brain imaging of pain: State of the art. J. Pain Res. 2016. doi:10.2147/JPR.S60433.

22 Henry DE, Chiodo AE, Yang W. Central nervous system reorganization in a variety of chronic pain states: A review. *PM R* 2011. doi:10.1016/j.pmrj.2011.05.018.

23 Fuchs PN, Peng YB, Boyette-Davis JA, Uhelski ML. The anterior cingulate cortex and pain processing. Front. Integr. Neurosci. 2014. doi:10.3389/fnint.2014.00035.

24 Hill KP, Palastro MD, Johnson B, Ditre JW. Cannabis and Pain: A Clinical Review. *Cannabis Cannabinoid Res* 2017. doi:10.1089/can.2017.0017.

25 Battista N, Di Tommaso M, Bari M, Maccarrone M. The endocannabinoid system: an overview. *Front Behav Neurosci* 2012. doi:10.3389/fnbeh.2012.00009.

26 Who. WHO Expert Committee on Drug Dependence. Delta-9-tetrahydrocannabinol. *World Health Organ Tech Rep Ser* 2018.

27 Lynch ME. Preclinical science regarding cannabinoids as analgesics: An overview. Pain Res. Manag. 2005. doi:10.1155/2005/169093.

28 Herkenham M, Lynn AB, Little MD, *et al.* Cannabinoid receptor localization in brain. *Proc*

Natl Acad Sci 1990; **87**:1932–6.

29 Lee MC, Ploner M, Wiech K, *et al.* Amygdala activity contributes to the dissociative effect of cannabis on pain perception. *Pain* 2013. doi:10.1016/j.pain.2012.09.017.

30 Weizman L, Dayan L, Brill S, *et al.* Cannabis analgesia in chronic neuropathic pain is associated with altered brain connectivity. *Neurology* 2018. doi:10.1212/WNL.0000000000006293.

31 Bloomfield MAP, Hindocha C, Green SF, *et al.* The neuropsychopharmacology of cannabis: A review of human imaging studies. Pharmacol. Ther. 2019. doi:10.1016/j.pharmthera.2018.10.006.

32 Whiting PF, Wolff RF, Deshpande S, *et al.* Cannabinoids for medical use: A systematic review and meta-analysis. JAMA - J. Am. Med. Assoc. 2015; **313**:2456–73.

33 Aviram J, Samuelly-Leichtag G. Efficacy of cannabis-based medicines for pain management: A systematic review and meta-analysis of randomized controlled trials. Pain Physician. 2017.

34 Russo EB. Taming THC: potential cannabis synergy and phytocannabinoid-terpenoid entourage effects. *Br J Pharmacol* 2011; **163**:1344–64.

35 Russo EB. The case for the entourage effect and conventional breeding of clinical cannabis: No “Strain,” no gain. *Front Plant Sci* 2019; **9**:1969.

36 Russo B, Guy G. A tale of two cannabinoids: the therapeutic rationale for combining tetrahydrocannabinol and cannabidiol. *Med Hypotheses* 2006; **66**:234–46.

37 Heuberger JAAC, Guan Z, Oyetayo OO, *et al.* Population Pharmacokinetic Model of THC Integrates Oral, Intravenous, and Pulmonary Dosing and Characterizes Short- and Long-term Pharmacokinetics. *Clin Pharmacokinet* 2015. doi:10.1007/s40262-014-0195-5.

38 Millar SA, Stone NL, Yates AS, O’Sullivan SE. A systematic review on the pharmacokinetics of cannabidiol in humans. Front. Pharmacol. 2018; **9**. doi:10.3389/fphar.2018.01365.

39 Huestis MA. Human cannabinoid pharmacokinetics. Chem. Biodivers. 2007; **4**:1770–804.

40 Karschner EL, Darwin WD, Goodwin RS, *et al.* Plasma cannabinoid pharmacokinetics following controlled oral Δ9-tetrahydrocannabinol and oromucosal cannabis extract administration. *Clin Chem* 2011; **57**:66–75.

41 Lucas CJ, Galettis P, Schneider J. The pharmacokinetics and the pharmacodynamics of cannabinoids. Br. J. Clin. Pharmacol. 2018; **84**. doi:10.1111/bcp.13710.

42 MacCallum CA, Russo EB. Practical considerations in medical cannabis administration and dosing. Eur. J. Intern. Med. 2018; **49**. doi:10.1016/j.ejim.2018.01.004.

43 de Hoop B, Heerdink ER, Hazekamp A. Medicinal Cannabis on Prescription in The Netherlands: Statistics for 2003–2016. *Cannabis Cannabinoid Res* 2018; **3.1**.

doi:10.1089/can.2017.0059.

44 Miller SL, Yeh HH. Neurotransmitters and Neurotransmission in the Developing and Adult Nervous System. In: *Conn's Translational Neuroscience*. , 2017. doi:10.1016/B978-0-12-802381-5.00004-X.

45 Manzanares J, Julian MD, Carrascosa A. Role of the Cannabinoid System in Pain Control and Therapeutic Implications for the Management of Acute and Chronic Pain Episodes. *Curr Neuropharmacol* 2006; **4**:239–57.

46 Torres-Moreno MC, Papaseit E, Torrens M, Farré M. Assessment of Efficacy and Tolerability of Medicinal Cannabinoids in Patients With Multiple Sclerosis: A Systematic Review and Meta-analysis. *JAMA Netw open* 2018. doi:10.1001/jamanetworkopen.2018.3485.

47 Yanes JA, McKinnell ZE, Reid MA, *et al.* Effects of Cannabinoid Administration for Pain: A Meta- Analysis and Meta-Regression. *Exp Clin Psychopharmacol* 2019. doi:10.1037/pha0000281.

48 Nielsen S, Sabioni P, Trigo JM, *et al.* Opioid-sparing effect of cannabinoids: A systematic review and meta-analysis. Neuropsychopharmacology. 2017. doi:10.1038/npp.2017.51.

49 Chelliah MP, Zinn Z, Khuu P, Teng JMC. Self-initiated use of topical cannabidiol oil for epidermolysis bullosa. Pediatr. Dermatol. 2018. doi:10.1111/pde.13545.

50 Johnson JR, Burnell-Nugent M, Lossignol D, *et al.* Multicenter, Double-Blind, Randomized, Placebo-Controlled, Parallel-Group Study of the Efficacy, Safety, and Tolerability of THC:CBD Extract and THC Extract in Patients with Intractable Cancer-Related Pain. *J Pain Symptom Manage* 2010. doi:10.1016/j.jpainsymman.2009.06.008.

51 Robson P. Abuse potential and psychoactive effects of δ-9-tetrahydrocannabinol and cannabidiol oromucosal spray (Sativex), a new cannabinoid medicine. Expert Opin. Drug Saf. 2011. doi:10.1517/14740338.2011.575778.

52 Wade DT, Makela PM, House H, *et al.* Long-term use of a cannabis-based medicine in the treatment of spasticity and other symptoms in multiple sclerosis. *Mult Scler* 2006. doi:10.1177/1352458505070618.

53 Zehra A, Burns J, Liu CK, *et al.* Cannabis Addiction and the Brain: a Review. J. Neuroimmune Pharmacol. 2018. doi:10.1007/s11481-018-9782-9.
